# Diversity, distribution, and population structure of *Escherichia coli* in the lower gastrointestinal tract of humans

**DOI:** 10.1371/journal.pone.0328147

**Published:** 2025-07-10

**Authors:** Rasel Barua, Paul Pavli, David Gordon, Claire O’Brien

**Affiliations:** 1 College of Health and Medicine, Australian National University, Canberra, Australian Capital Territory, Australia; 2 Research School of Biology, Australian National University, Canberra, Australian Capital Territory, Australia; 3 Inflammatory Bowel Disease Research Laboratory, Canberra Hospital, Canberra, Australian Capital Territory, Australia; 4 Gastroenterology and Hepatology Unit, Canberra Hospital, Canberra, Australian Capital Territory, Australia; 5 Centre for Research in Therapeutic Solutions, Faculty of Science and Technology, University of Canberra, Canberra, Australian Capital Territory, Australia; Texas A&M University College Station: Texas A&M University, UNITED STATES OF AMERICA

## Abstract

Several studies report the diversity, and population structure of *Escherichia coli* (*E. coli*) in the human gut, but most used faecal specimens as the source of *E. coli* for analysis. In the present study, we collected mucosal biopsies from three different locations: the terminal ileum, transverse colon, and rectum from 46 individuals. To identify unique strains, we fingerprinted about 3300 isolates of *E. coli* via the multiple-locus variable-number tandem-repeat analysis (MLVA) technique. An example of each strain per individual then underwent PCR for phylogrouping, and specific phylogrouped strains were further screened to determine whether they belonged to one of four common human-associated sequence types (ST69, ST73, ST95, and ST131), and to identify B2-subtypes. We detected on average 2.5 unique strains per individual. The frequency of unique strain(s) appeared in individuals as follows: 35% (16/46) had only one strain, 22% (10/46) had two strains, 24% (11/46) had three strains and 4% (2/46), 9% (4/46) and 7% (3/46) had 4, 5 and 6 strains, respectively. Strain richness did not depend on gender, age, or disease status. The most abundant phylogroup in all gut locations was B2 followed by A, B1, and D. Strain richness overall and across gut locations was decreased if an individual’s dominant strain belonged to phylogroup B2. ST95, ST131, and ST73 constituted more than half of the total B2 strains. Analysis of B2 sub-types revealed that sub-types IX (STc95) and I (STc131) were more common than other sub-types. The phylogroup and ST of strains at different gut locations did not vary significantly. However, there were multiple examples of individuals who carried strains detected only in one gut location. The present study suggests that particular phylogroups and STs are likely to dominate in different locations in the lower gut of humans.

## Introduction

The distribution and population structure of *Escherichia coli* (*E*. *coli*) in humans and other non-human hosts are reported in several studies using faecal samples [1–6], but there is a lack of information concerning the distribution of *E. coli* along the gut of humans. Although our research group previously demonstrated the diversity of *E*. *coli* population structure in the human lower gut [7], the present study attempts to further explore the diversity and distribution of *E. coli* at both the population and sub-population levels.

*E. coli*, a member of *Enterobacteriaceae*, commonly exist as commensals in the intestines of humans and other warm-blooded mammals [8]. Some host factors, for example, the morphology of the intestine, size, sex, and age may influence the detection of *E. coli* in hosts [1,9]. Due to high genome plasticity and a massive collection of genes, the population structure of *E. coli* is genetically diverse [10,11].

*E. coli* strains are classified into major phylogroups: A, B1, B2, and D, and minor phylogroups: C, E, F, G, and H [8,12]. Phylogroup B2 is commonly recovered from the gut of people living in developed countries, such as Australia [2,7]. Phylogroups B2 and D contain notable sequence types (STs), such as ST73, ST95, ST131, and ST69, which are associated with extra-intestinal infections in humans [13]. Strains may be superior colonizers of the human gut if they possess specific factors, for example, higher expression of P or S fimbriae, and the presence of a K5 capsule in B2 strains, which makes them more persistent colonizers than other phylogroups [14]. Also, diet and gut dynamics may influence the strains’ abundance in the gut [15–17].

*E. coli* inhabit mainly in the lower gut of humans and other mammals [18–21]. The lower gut is composed of several regions: the ileum, colon, and rectum [22,23]. These regions vary in their physiological characteristics, such as pH, nutritional flow, inorganic salt concentrations, epithelial cell types, and others [22,24–26]. In mammals – for example, pigs – the isolation of *E. coli* strains and their characteristics, such as the frequency of detection of different types of colicin genes from those isolates, have been found to vary from region to region of the intestinal tract [27]. Moreover, in a recent study, we showed that *E*. *coli* strains may exhibit variations at the genomic and phenotypic level when they are collected from two different locations of the human lower gut [28].

Given the variation in morphological and biochemical properties of different gut locations and their influence on strain colonization, we isolated *E. coli* from biopsy samples collected from multiple locations rather than faecal samples. We used these to determine the location-specific distribution and population structure of commensal mucosa-associated *E. coli*. Our previous work examined the diversity of *E. coli* from five locations in the human gut, but biopsies were collected mostly from individuals suffering from inflammatory bowel disease (IBD) (normal – 26% and IBD – 74%) [7]. Moreover, that study lacked data at the sub-population level, such as STs and B2 sub-lineages.

The present study aimed to determine the diversity, distribution, and population structure of *E. coli* in the lower gut of healthy humans and used mucosal biopsies from three different locations. Over 3300 *E. coli* isolates obtained from the terminal ileum, transverse colon, and rectum of 46 individuals were characterised to obtain a more precise understanding of the population structure of *E. coli* in the human gut.

## Materials and methods

### Ethics statement

The Australian Capital Territory (ACT) Health Human Research Ethics Committee (HREC), Research Ethics and Governance Office, Health Directorate, ACT Government, Australia, approved the present study (project reference number- ETH.5.07.464). Before the sampling, the individuals were informed about the collection of biopsy specimens and written consent was obtained.

### Biopsy sampling and collection of isolates

Biopsy sampling was carried out by designated gastroenterologists during routine colonoscopy originally from 50 individuals in the period between September 19 and November 27, 2019, at the Gastroenterology and Hepatology Unit (GEHU), Canberra Hospital, ACT, Australia. Four samples that did not show *E. coli* growth were excluded from the analysis. Subjects were prepared for colonoscopy with the ingestion of 2–3 L of polyethylene glycol. The superficial mucous layer was washed away with a jet spray and the biopsy channel was flushed with sterile water. Using sterile pinch forceps, samples were collected from the rectum, transverse colon, and terminal ileum, in that order. Individuals’ indication for colonoscopy and medications during the procedure have been provided in the supplementary information (S1 Table). On average, 2–3 biopsies were collected from each location. Not all three sets of biopsies were collected from all individuals, and this information is provided in the supplementary information (S2 Table). A 15 mL polypropylene tube containing 1 mL of lysogeny broth (LB) was used to store each set of biopsies and the samples were transported to the laboratory at room temperature.

The samples were incubated overnight with continuous shaking at 150 rpm at 35.5ºC, and dilution streaked onto MacConkey agar media (Neogen) to isolate single colonies [29]. Colonies showing both lactose-positive and lactose-negative growth characteristics were selected and incubated overnight at 35.5ºC on LB and Simmons citrate (SC) agar plates. We aimed to isolate 31 single colonies from each dilution plate from the terminal ileum, transverse colon, and rectum of each subject, but could not retrieve the total 93 single colonies for some individuals (S2 Table). Each colony was given an identification (ID) number and treated as a single ‘isolate’. For further analysis, we selected only those isolates that showed negative growth on Simmons citrate agar, consistent with *E*. *coli*.

### Identification of unique strains

We used multiple-locus variable-number tandem-repeat analysis (MLVA) with the minor modifications described by Camelena *et al.* [30] for the identification of unique strains in each gut location. The conventional boiling method was used to retrieve DNA from *E. coli* cells. The PCR was carried out in a 25 µL reaction volume in a single tube using the Qiagen multiplex PCR kit (Qiagen). The PCR components were 3.75 µL of total primers (each 10 µM primer, Sigma Aldrich), 12.5 µL of PCR master mix, 2.5 µL of Q solution, 3.75 µL of nuclease-free water, and 2.5 µL of supernatant as DNA template. The PCR program included a denaturation step of 95°C for 15 min, followed by 30 cycles of denaturation at 94°C for 30 s, annealing at 55°C for 90 s, and an extension step of 72°C for 90 s as well as a final extension of 72°C for 10 min. The PCR products were run in a 3% agarose (Bioline) gel stained with ethidium bromide for 75 minutes at 100 volts (V). The gels were visualised using a Gel Doc^TM^ XR+ imaging system (Bio-Rad). The same imaging protocol was used whenever required. We recorded one example of each unique isolate for each gut location: terminal ileum, transverse colon, and rectum, for each patient. We preserved each unique isolate in glycerol (glycerol: culture = 2: 5, v/v) at −80ºC.

### Phylogroup determination, profiling extra-intestinal linked four major sequence types (STs), and sub-typing B2 strains

To identify phylogroups and other molecular profiles, we extracted bacterial genomic DNA using DNAzol^®^ Genomic DNA Isolation Reagent (Molecular Research Center Inc.). The DNA was precipitated in ethanol and collected in a “TE buffer-NaOH” mixture. Each example of unique strains isolated from different gut locations that were suspected to be the same, run side-by-side on another MLVA gel to re-confirm that the strains were indeed the same. If isolates of the ileum, colon, and rectum of an individual showed similar MLVA band patterns, then those isolates were considered to be examples of a similar unique strain of that individual. We selected only one representative of each unique strain per individual for further analysis.

We used the quadruplex Clermont phylo-typing PCR method [31] which is specific for determining the phylogroups: A, B1, B2, C, D, E, F, and *Escherichia* clades. As the Clermont phylo-typing method classifies only the traditional phylogroups, phylogroups G and H are therefore missing from our analysis. Several strains showed bands potentially indicating both phylogroups A and C. In such cases, specific primers were used to distinguish between phylogroups A and C. Similarly, by using specific primers, we also distinguished the isolates that showed band patterns potentially indicating either phylogroup D and E, or E and *Escherichia* clade I.

We used the PCR method described by Doumith *et al.* [32] for profiling four major human-associated extra-intestinal STs: ST69, ST73, ST95, and ST131, and only representative isolates of B2 and D phylogroups were selected for profiling. To determine the B2 sub-types, all isolates assigned as B2 phylogroup were analysed with the PCR protocol described by Clermont *et al.* [33].

### Statistical analysis

All data were analysed in Microsoft Excel and in the statistical software package JMP v.15 (2019, SAS Institute Inc.). Pearson’s chi-squared test was performed to compare nominal variables in different groups. The fit model analysis of variance (ANOVA) test was carried out to compare means among different parameters, such as an individual’s age, gender, disease status, and strain richness. The analysis of means (ANOM) test was used to compare means between the two groups. Analysis that revealed an α value of 0.05 was considered statistically significant (default 95% confidence interval, p ≤ 0.05).

## Results

### Diversity of *Escherichia coli* among-individuals

**Fig 1:**
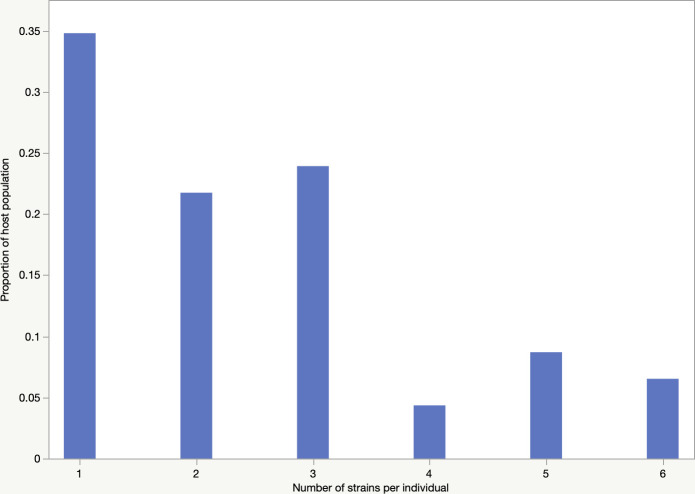
Frequency of detection of unique strains in different individuals.

From 46 individuals, we retrieved a total of 3355 *E*. *coli* isolates (single colonies), which we analysed using the MLVA fingerprinting method to identify unique strains within each individual. The average number of unique strains per individual was 2.5 (± 0.23 S.E., range 1–6; Fig 1). A single strain only was present in 35% (16/46) of individuals, followed by two in 22% (10/46) and three in 24% (11/46) of individuals, respectively. More than three strains were found in fewer individuals: 4 in 4% (2/46), five in 9% (4/46), and six in 7% (3/46), respectively. A detail on the number(s) of the identified unique strain(s) for each individual has been provided in S2 Table. The frequency of unique strains was independent of gender, age, and disease state of the individuals (ANOVA: Prob>F = 0.54). Metadata for the individuals, including age, sex and disease condition, are provided in S2 Table. We had information about antibiotic exposure within six months of the colonoscopy for 43/46 individuals (S2 Table). We found that the richness or frequency of strains was not dependent (p = 0.2637) on antibiotic exposure (14 individuals exposed and 29 not exposed) (but also see below).

**Fig 2:**
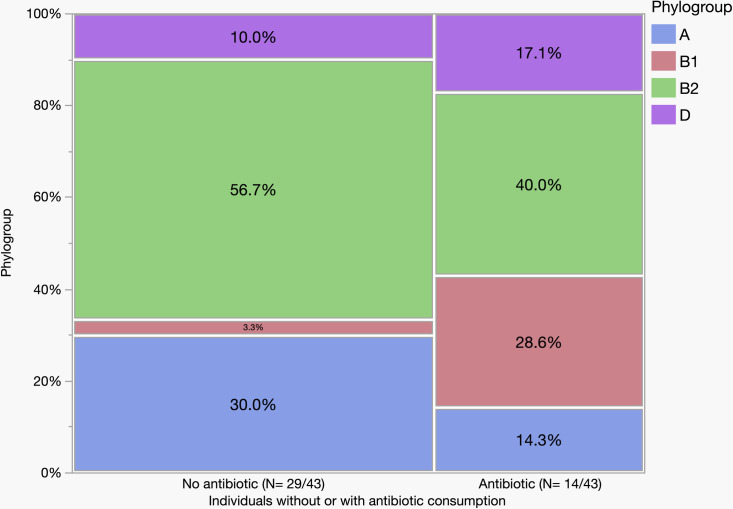
Abundance of phylogroups A, B1, B2, and D in two groups of individuals who did not (N = 29) and did (N = 14) consume antibiotics within six months at the time of biopsy sampling. The graph represents an analysis of the unique strains and their phylogroup for strains obtained from 43/46 individuals.

The phylogroup was determined for each isolate in each gut location and further analyses were conducted to determine their distribution. The isolates were assigned as phylogroup B2 - 49%; A – 18%; D – 12%; B1 - 12%; F – 6%; E – 2%; and *Escherichia* clade I – < 1% (S1 Fig and S3 Table for details). None of the studied isolates were found to belong to phylogroup C. Phylogroup B2 strains were present in 74% of individuals. Other phylogroups were found at different frequencies in the individuals: phylogroup A – 33%; D – 26%; and B1 - 20% (S2 Fig). Fewer individuals carried the minor phylogroups – phylogroup F – 9%; E – 7%; and *E*. clade I – 2%. Out of 43 individuals who provided information on antibiotic consumption, 14 individuals were exposed, and 29 individuals were not. These groups had significant variation in the carriage of the four main phylogroups – A, B1, B2 and D (p = 0.0014). Individuals who did not receive antibiotics carried a higher number of phylogroup B2 and A strains (Fig 2, with details in S2 and S3 Tables).

**Fig 3:**
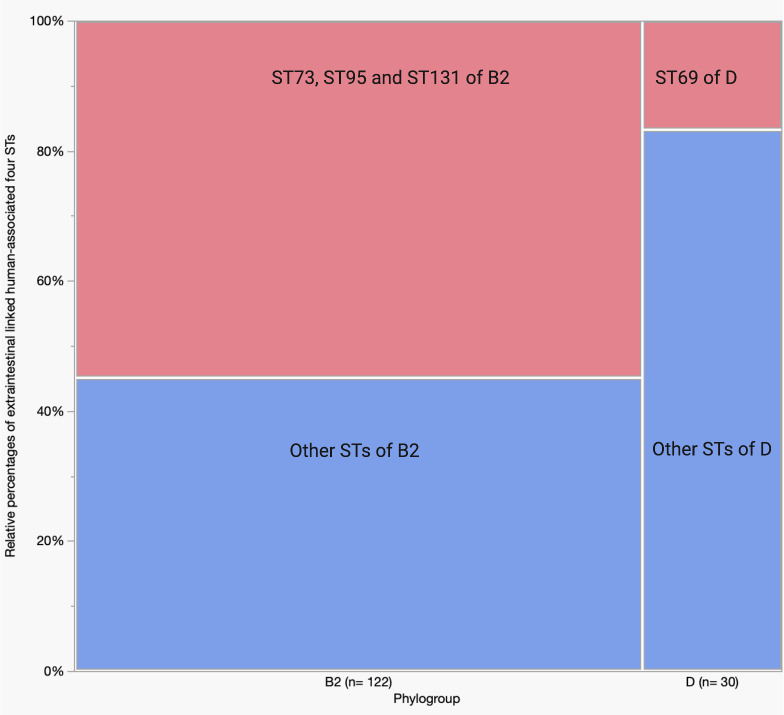
Relative abundance of the four major sequence types of phylogroup B2 and D strains. A total of 152 representative isolates of phylogroup B2 and D were analysed.

**Fig 4:**
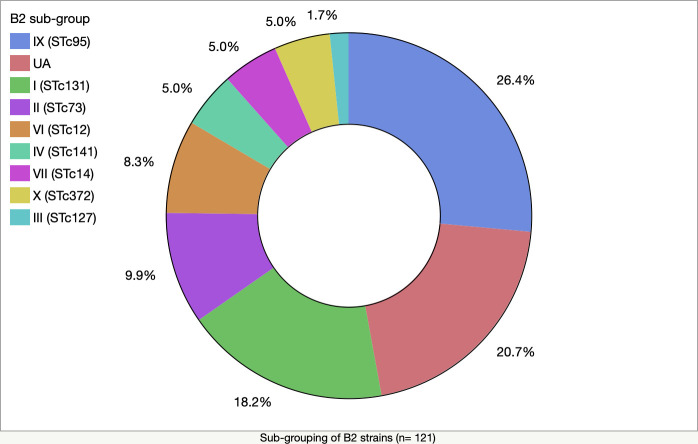
Abundance of B2 sub-lineages for the 121 phylogroup B2 strains.

Phylogroup B2 and D were further analysed for four STs: ST73, ST95, ST131, and ST69, and B2 strains were assessed for their sub-lineages. Of the total B2 strains, ST73, ST95, and ST131 accounted for 55% of the total B2 isolates (ST95–25%, ST131–21%, and ST73–9%) (Fig 3 and S3 Table). Of the D strains, 17% belonged to ST69 (Fig 3 and S3 Table). During the analysis of B2 sub-types, we excluded a single strain that failed to show bands despite multiple gel electrophoresis runs. Sub-types IX (STc95), and I (STc131) appeared more abundantly than other sub-lineages, such as II (STc73), VI (STc12), IV (STc141), VII (STc14), X (STc372), and III (STc127) (Fig 4 and S3 Table). More than 20% of B2 isolates were unassignable (UA).

### Diversity of *Escherichia coli* among gut regions

The richness and diversity of *E. coli* strains were analysed in each gut location (Table 1). The mean number of strains detected in a region was defined as strain richness. We excluded those individuals who carried only a single strain. Analysis based on individuals’ gender, age, or disease condition, revealed that strain richness did not vary by gut location or within locations.

**Table 1:** Determination of *E. coli* strain richness and diversity in the three gut regions examined.

Gut region	Strain richness (mean number of strains per region) ± SE	Diversity (Simpson’s index of diversity) ± SE
Ileum	2.33 ± 0.185	0.973 ± 0.027
Colon	2.41 ± 0.176	0.974 ± 0.026
Rectum	2.52 ± 0.214	0.973 ± 0.028

Individuals were excluded from the analysis if only a single strain appeared in all three gut regions. Each gut region included the following number of individuals, ileum, n = 27; colon, n = 29; and rectum, n = 29 (details of the data are provided in S3 Table).

We used Simpson’s diversity index (SDI) to calculate the strain diversity [34,35]:


1−Σn(n−1)/N(N−1)\]

In this equation, n represents the number of strains detected in each gut region of all subjects, and N represents the sum of strains in each gut region of an individual. Although all three regions showed similar values of diversity (Table 1), a high SDI indicates all locations carried diverse strains (maximum SDI value is 1).

**Fig 5:**
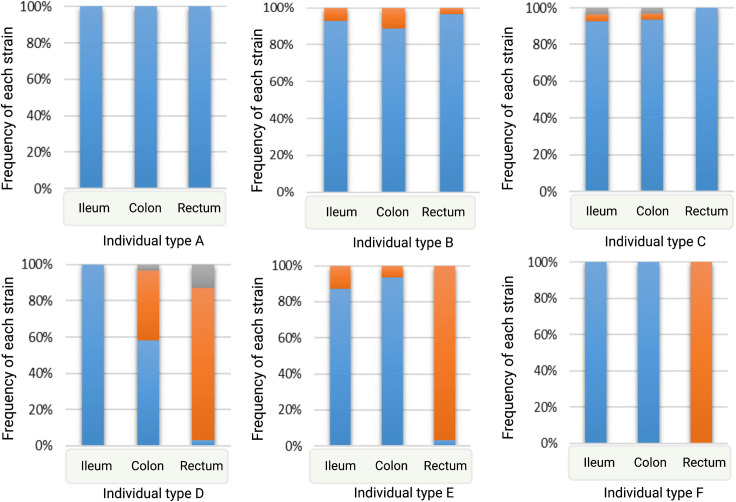
Different patterns of strain distribution in the three gut regions. A strain is indicated by a colour in a bar and the same colour across the regions indicates the distribution of the same strain. The graphs were generated by analysing the frequency of unique strain(s) in each gut region for each individual. Individual type A: Blue colour in all the gut regions indicates that only a single strain was found across these regions. Individual type B and C: The most frequent unique strain found in all regions is indicated by blue, while orange and grey indicates minor unique strains. Individual type D and E: A single unique strain appeared more frequently in either two or one region, as indicated by blue and orange, respectively. Individual type F: The unique strain that was found only in the ileum and colon, indicated by blue, disappeared in the rectum; instead, a different unique strain appeared, indicated by orange. Of the 46 individuals, type A was found in 16, type B and C in 18, type D and E in 5, type F in 1, and the extended version of type B and C or D and E in 6 individuals.

Each gut location also revealed various patterns of strain distribution (Fig 5). The patterns are mainly as follows: i) all regions carried a single and the same strain (individual type A), ii) all regions were dominated by a particular strain and other strain(s) appear as minor (individual type B and C), iii) one or two regions were dominated by a specific strain (individual type D and E), iv) one region was found to exclusively carry only a single strain (individual type F), and v) two or all the three regions were dominated at a similar frequency by at least two strains and other strains either appeared at a lower frequency or disappeared from one or two locations (not shown in Fig 5). A detail on individual types carrying various patterns of strain distribution has been provided in S3 Table. The latter type is more likely an extended version of individual type B and C or D and E. Analysis of strain distribution in individuals based on antibiotic use showed individuals who did not take antibiotics were more frequent in most of the observed patterns. The distribution patterns for antibiotic use (antibiotic versus non-antibiotic) appeared as follows: individual type A- 5 vs. 10, individual type B and C- 6 vs. 10, individual type F- 0 vs. 1, and the extended version of individual type B and C or D and E- 0 vs. 6. Despite this trend, the opposite was observed for individual type D and E, where the number of individuals who consumed antibiotics was relatively higher (3/5). As mentioned earlier, the number of individuals who did not consume antibiotics (N = 29) was twice the number of individuals who did (N = 14). We excluded individuals from the analysis for whom antibiotic use information was unavailable (N = 3).

**Fig 6:**
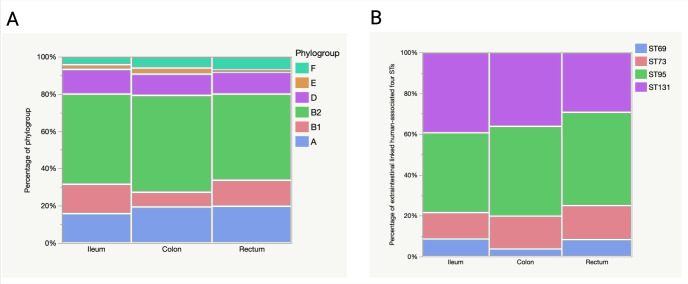
Relative abundance (percentage) of the phylogroups and four major STs in the three gut regions. A. For the phylogroups, we analysed a total of 251 genotypes detected in each gut region of 46 individuals. B. For analysis of the ST73, ST95, ST131 and ST69, we analysed all 152 representatives of the B2 and D strains.

The distribution of phylogroups and four major STs were analysed for significant variations along the gut regions. Although all the regions were dominated by phylogroup B2 strains, there was no significant variation of each phylogroup distribution among the gut regions (p = 0.8968) (Fig 6A). Also, the three regions did not vary significantly in their ability to harbor four STs: ST95, ST131, ST73, and ST69 which are associated with extra-intestinal infections (p = 0.9825) (Fig 6B).

### Interactions among phylogroups within individuals

**Fig 7:**
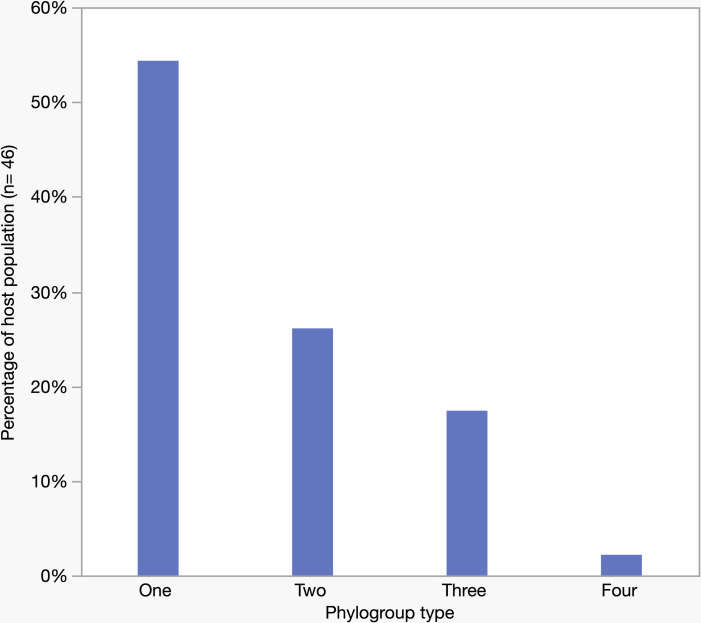
Number of phylogroups occurring in individuals.

A single phylogroup was detected in more than half of all individuals (25/46) (Fig 7). In contrast, strains belonging to two and three phylogroups appeared in about one-fourth (12/46) and one-fifth (8/46) of the individuals, respectively. Only one individual was found to carry four phylogroups, and more than four phylogroups was not detected in any individual. Focussing only on those individuals who carried two phylogroups, it is hypothetically possible to observe 15 different combinations of two phylogroups (out of six). According to the combinatorial formula, nCr = nPr/r! = n!/r!(n-r)! where n = 6 (total phylogroups), r = 2 (co-existence of two phylogroups). However, the co-existence of two phylogroups in one-fourth of individuals was restricted to only four combinations: A-B2, B1-B2, B2-F, and A-D (S3 Fig). A greater number of combinations was found in individuals who carried three or four phylogroups. However, we did not find any the co-existence of D and F, or B1 and F, in any individuals who harboured two, three, or four phylogroups.

**Fig 8:**
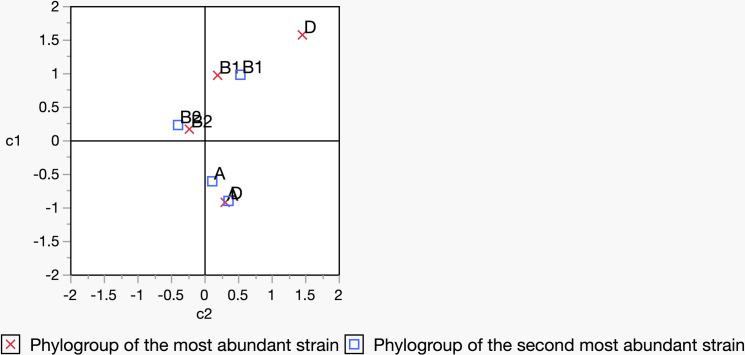
The relationship between the most (marked in a red ‘X’) and second most abundant strain (marked in a blue square box) belonging to the same phylogroup. The clustering of B2 with a red cross and B2 with a blue square box indicates that if the most abundant strain belongs to phylogroup B2, the second most abundant strain will likely belong to phylogroup B2. A similar pattern is also likely to appear when the most abundant strain belongs to phylogroup B1, but no such pattern was observed when the most abundant strain belongs to phylogroup A and D. In addition to this figure, a contingency table has been provided in the supplementary data (S4 Table) to demonstrate the relationship further.

There was a tendency, when individuals carried two or more strains, that the second most abundant strain was more likely to belong to the same phylogroup as the most abundant strain. This scenario was more likely to occur if the most abundant strain belonged to phylogroup B2 (Fig 8 and S3 Table), although an overall analysis did not reveal any such significant relationship when a correspondence analysis was carried out among the four major phylogroups: A, B1, B2, and D (p = 0.1017).

**Fig 9:**
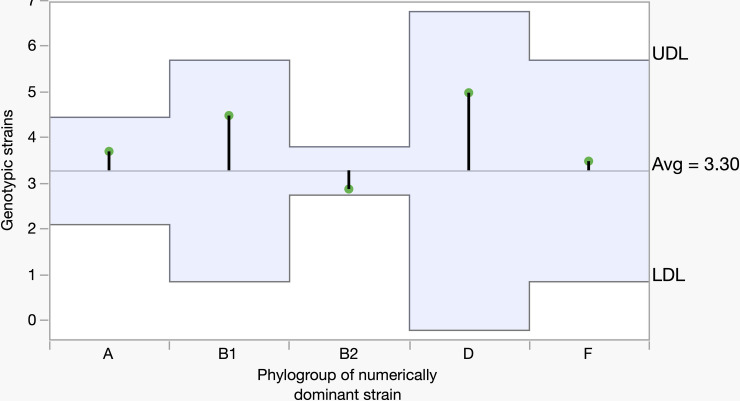
The relationship between the phylogenetically dominant strain and average number of genotypes per individual. The x-axis shows the phylogroups of the numerically dominant strains, and the y-axis shows the average number genotypic strains. The green tip indicates the average genotype value against each dominant phylogroup. The upward and downward green tips indicate values greater than and lower than the average (Avg. = 3.30), respectively. The variations in the upper decision limit (UDL) and lower decision limit (LDL) for different phylogroups of the numerically dominant strain are due to differences in sample size. All the values appeared in green tip and fell within the decision limits indicates no significant variation from the overall average value. Otherwise, the values that significantly differ from the average would appear with a red tip and fall outside the decision limits.

The relationship between the phylogroup of the dominant strain and the average number of genotypically diverse strains was determined. The analysis included only those individuals who carried at least two or more strains. We found the least number of genotypic strains when the dominant strain belonged to phylogroup B2 (Fig 9). In contrast, the number of genotypic strains was found to be relatively higher than the average when the dominant strain belonged to phylogroups A, B1, D, and F. It is noteworthy to mention that individuals carrying phylogroups other than B2 were smaller in numbers: dominant strains belonging to phylogroup A appeared in seven individuals, B1 in two, D in one, and F in two individuals. However, an overall analysis did not reveal any significant relationship between the dominant strain and average number of genotypes (p = 0.2339).

## Discussion

There are several differences between the present study and another similar study done by our group (Gordon *et al.* [7]). Almost 35% of individuals carried a single strain and no individuals carried more than six strains. In contrast, Gordon *et al* found only 15% of individuals carried a single strain and some individuals carried seven or eight strains (S5 Table). The average number of unique strains detected in the present and previous studies were 2.5 and 3.5, respectively. Although mucosal biopsies were used as the source of *E. coli* isolates in both studies, the variation in number of individuals harboring unique strain(s) may be due to variations in the number of study subjects and gut locations. The other study included 33% more subjects and used additional biopsies from the ascending and descending colons.

The average number of unique strains of *E. coli* in the human gut has been reported in several studies [1,2,7,36–38]. Based on an analysis of faecal specimens, the average number of unique strains per individual was reported as being around 1–2 which is lower than that observed in the present study [2,36]. Another study reported recovery of almost 2.7 genotypes from stool samples collected from Gambian children [39].

Geographical location may also be an important factor, for example, Foster-Nyarko *et al* in their study on Gambian children found only 9% of strains belonged to phylogroup B2 [39]. Previous studies reported an association between the geographical location and distribution of phylogroups: people living in temperate and developed countries carry mainly phylogroup B2 strains, whereas people living in tropical and developing countries carry more phylogroup B1 strains [40,41]. The present study also revealed an abundance of B2 strains followed by A, D, and B1 phylogroups. A similar outcome was also observed in previous studies of the same population (Canberra, Australia) [1,2,7]. In addition, a significant variation was observed in the distribution of the four major phylogroups between individuals who did (14/43) and did not (29/43) receive antibiotics within six months of the time of colonoscopy.

Three human-associated STs: ST73, ST95, and ST131, when combined, appeared more commonly than other STs of the B2 phylogroup. In contrast, only about 15% of the total D phylogroup strains were ST69. The recovery of all four STs in humans was previously reported in multiple studies [42–45]. Bourne *et al.* [3] referred to unpublished data that these four STs were found to constitute about one-third of the total *E. coli* isolates in faecal specimens of people living in the Canberra region. Consistent with our observations, Dixit *et al.* [46] recovered 35 genotypes of D phylogroup from 67 patients, and found 14% were ST69 and 77% non-ST69 strains (STs were not provided for 9% of D phylogroup strains). Sub-typing of B2 strains also reflected the distribution of ST73, ST95, and ST131. Sub-types IX (STc95), I (STc131), and II (STc73) combinedly constitute almost 55% of B2 sub-types. Other B2 sub-types identified were VI (STc12), IV (STc141), VII (STc14), and III (STc127) which were also reported in clinical and commensal isolates from humans [47,48]. However, humans infected with urinary tract infections (UTIs) were found to carry certain STs, such as ST69, ST73, ST95, and ST131 [42,49–51]. Gut bacteria may have an association with UTI [18,52], and in two independent studies, strains recovered from urine were the dominant clones in faecal specimens of 55% (6/11) and 71% (30/42) of women suffering from acute cystitis [53,54]. Therefore, our study suggests that the abundance of ST73, ST95, and ST131, which usually reside as commensals in the gut, may pose a risk to humans as the source of extra-intestinal infections.

Our study demonstrates that in some cases, especially when the most abundant strain is from the B2 phylogroup, there is a propensity for the second most abundant strain to be from the same phylogroup, although the overall analysis did not reveal a significant outcome (Fig 8). A total of 28 individuals was included in the analysis, where B2 was most abundant in 18 individuals followed by A in 7, B1 in 2 and D in one individual. A pattern of the most abundant strain having the same phylogroup as the second-most abundant strain has been demonstrated previously by Gordon *et al.* [7]. Within-host genomic evolution of strains possibly contributes to the cases where the most and second most abundant strains belong to the same phylogroup [46]. However, other factors may also contribute to such an association, for example, bacteriocin secretion [55,56], nutrient uptake competition [57], and phage-bacterium interaction where strains of the same phylogroup may show resistance or sensitivity to certain phages [58].

The individuals who were admitted for colonoscopy were the source of biopsy specimens for this study. The individuals may have had underlying disease conditions (e.g., iron deficiency and inflammatory bowel disease) or been on medication (S1 Table) that might influence the microbiome profile [59–64], and this may limit the strength of the present study. Collecting biopsies solely for such a study would not be ethical as colonoscopy involves an invasive procedure. Another possible limitation is the use of single biopsy forceps for each individual, but we collected biopsies sequentially from the rectum, colon, and ileum which is microbiologically a rational practice to avoid location-specific microbial contamination. Nutritional flow in the gut is directed from the ileum to the colon to the rectum [65], but forceps first come in contact with the rectum during colonoscopy. Therefore, our sampling strategy was designed to overcome this limitation. Moreover, the strength of this study is the use of biopsies instead of faecal specimens, which has allowed us to look at location-specific strain distribution and their population structure.

In summary, this study suggests that the population structure of *E. coli* and their distribution in the lower gut of humans does not occur randomly. The most abundant and well-adapted phylogroup is B2, and its presence as a dominant strain may generate the least genotypic heterogeneity in the gut, although it co-exists with other phylogroups. Some major B2 sequence types: ST73, ST95, and ST131 are more commonly found in the gut which indicates that the gut may be a potential reservoir for extra-intestinal infections in humans (e.g., urinary tract). We failed to identify any significant variation in the distribution of phylogroups and major sequence types among the gut regions, but strain distribution at the individual level reveals an indication of location-specific strain colonization. In the future, it would be worth studying if the genetic content of the four major STs (ST69, ST73, ST95, and ST131) vary when they are isolated from urine and faecal samples of both healthy and UTI individuals. Such a study will help to understand the changes in the genetic nature of those strains in two different environments during urinary tract infections in humans. As the present study also revealed that individuals who carried two, three or four phylogroups did not show co-existence of phylogroups D and F or B1 and F, it would be noteworthy to conduct comparative phenotypic studies followed by genomic analysis of strains between D and F or B1 and F. This may help to understand whether strains belonging to phylogroup F compete with strains belonging to phylogroup D or B1.

## Supporting information

S1 Table Individual’s indication for colonoscopy and ongoing medication during colonoscopy. (DOCX)

S2 Table Individual’s demography, disease status, antibiotic consumption, sampled gut regions, retrieved total isolates and genotype(s). (DOCX)

S3 Table Frequency of unique strains isolated from respective gut regions and their classification into phylogroups, four major STs, B2 sub-types, and individual types based on strain distribution. (DOCX)

S4 Table Cell χ2 value showing the relationship between the phylogroup of the most and the second most abundant strains in an individual. (DOCX)

S5 Table A comparison between the current study and the study by Gordon *et al.* (2015) regarding the percentage of individuals carrying different number of unique strain(s). (DOCX)

S1 Fig Percentage of different phylogroups in the total isolate collection. For example, a total of 251 unique strains were detected across different gut regions of 46 individuals, of which 122 were identified as phylogroup B2 strains, accounting for approximately 49% of the total.
(TIF)

S2 Fig Percentage of individuals harbouring different types of phylogroup strains. A total of 46 individuals were studied. For example, phylogroup B2 strains were retrieved from 34 individuals, accounting for approximately 74% (73.91%) of the total individuals.
(TIF)

S3 Fig Co-existence of two phylogroups in individuals. The combinations were found restricted to A-B2, B1-B2, A-D, and B2-F.
(TIF)
