# Supplementary material for: Diversity, distribution, and population structure of Escherichia coli in the lower gastrointestinal tract of humans
Source: PLoS One. 2025 Jul 10;20(7):e0328147. doi: 10.1371/journal.pone.0328147 (PMC12244825; doi:10.1371/journal.pone.0328147)
Supplement: S1 Table — (DOCX) [file pone.0328147.s001.docx]

S1 Table. Individual’s indication for colonoscopy and ongoing medication during colonoscopy.

| Individual | Indication of colonoscopy | Ongoing medication during colonoscopy |
| --- | --- | --- |
| 1 | Polyarthralgia, alcoholic liver disease, haemorrhoids-internal, Peyronie's disease | - |
| 3 | Thickened transverse colon/ sigmoid diverticulitis | Telmisartan, Lipidil, Rosuvastatin, Allopurinol*,* Arthrexin |
| 4 | - | - |
| 5 | Positive FOBT (faecal occult blood test) | Ibuprofen, Panadol Osteo, Somac, Senna, Oemizon, Eleuphrat cream |
| 7 | Augmentin forte | Aricept, Baricitinib*,* Celebrex*,* Metformin*,* Endone, folic acid, Karvea*,* Lipitor*,* methotrexate*,* Nexium, Sulfasalazine*,* Sertraline |
| 8 | Rectal bleeding | Atacand (tablet), Proctosedyl (ointment) |
| 9 | Bowel cancer | Apixaban, Atorvastatin, vitamin D, Ventolin, Flomasta |
| 11 | Family history of colon cancer, abdominal pain | No medication |
| 12 | Ion deficiency, Hemochromatosis | Metformin, Norvasc, phenytoin, Micardis, Cardio Aspirin, Sertraline*,* Lamotrigine |
| 13 | Positive faecal occult blood test (FOBT) | No medication |
| 14 | Positive faecal occult blood test (FOBT) | Candesartan |
| 15 | Ruptured appendix | No medication |
| 16 | Ulcerative colitis | Zoloft |
| 17 | PR bleeding | Nexium, Antihistamines |
| 18 | Family Hx follow up | Lipitor 20 mg |
| 19 | Iron deficiency | Methadone, Verapamil |
| 20 | Anaemia, bleeding PR, renal transplant patient | Aranesp, Allopurinol, Labetalol, Lanix, Lipitor, Mycophenolate*,* Prednisone, Prolia*,* Ramipril*,* Somac*,* Tacrolimus |
| 21 | Crohn's disease | Infliximab, Ventolin, Sevitide, IVD |
| 22 | Colonic polyps | Dabigatran Etexilate*,* Candesartan cilexetil*,* Metoprolol*,* Digoxin, *G*limepiride*,* Empagliflozin*,* Metformin*,* Atorvastatin*,* Insulin glargine*,* Duloxetine Hydrochloride*,* Fenofibrate*,* Pantoprazole*,* Glucosamine Sulfate*,* Multi-vitamin, Cyanocobalamin, vitamin D, Calcium, vitamin D3, Broad spectrum probiotic |
| 23 | Iron deficiency and anaemia | Rabeprazole*,* Amoxycillin |
| 24 | Positive faecal occult blood test (FOBT) | Sacubitril*/*valsartan medication for diabetes |
| 25 | Rectal bleeding | Cymbalta, Candesartan |
| 26 | Positive faecal occult blood test (FOBT) | Vitamins |
| 27 | Crohn's disease | Infliximab infusion, Ventolin |
| 28 | NBCSP Positive faecal occult blood test (FOBT) | Micardis |
| 29 | Family history colorectal cancer | Methotrexate*,* Folic acid, Leflunomide |
| 30 | Iron deficiency | No medication |
| 31 | Follow up with polyps | Perindopril, Sitagliptin, Nexium, Panadeine Forte*,* Stemetil*,* Diazepam*,* Simvastatin |
| 32 | Iron deficiency, anaemia | No medication |
| 33 | Crohn's disease | Mobic*,* Salofalk*,* Lovan, Seroquel*,* Megafol |
| 34 | Positive faecal occult blood test (FOBT) | Coversyl |
| 35 | Exclusion of ulcer of the GI tract | Fish oil, vitamin D, Testosterone 14 weeks injection |
| 36 | Positive faecal occult blood test (FOBT) | Coversyl*,* Viagra |
| 37 | Iron deficiency, anaemia | Rebisol, Antistatin, Salbutamol |
| 38 | Crohn's disease | Gastro-Stop, Infliximab |
| 39 | Follow up with polyps | Nexium |
| 41 | Family history of colon cancer, has polyps | Levothyroxine, Voltaren*,* Clexane*,* Nurofen*,* Mobic, Vitamin D |
| 42 | PR bleeding | No medications |
| 43 | NBCSP Positive faecal occult blood test (FOBT) | Metoprolol*,* Apixaban (stopped three days ago), Seretide*,* Champix*,* Sertraline*,* Vig, Aorin |
| 44 | Colon cancer screening, family history of 1st degree relative with colorectal cancer, personal history of colonic polyps | Pevindopnil |
| 45 | Family history of colon cancer | No medication |
| 46 | Diverticulitis | Allopurinol |
| 47 | Constipation | Ramipril, Stalin |
| 48 | Rectal bleeding | Nexium, Fluoxetine*,* Mirtazapine |
| 49 | Family history of hereditary non-polyposis colorectal cancer, lunch like (non-predictive test) | No medication |
| 50 | Chronic diarrhea, exclusion of CD and UC | contraceptive |

“-”: data not available, NBCSP: National Bowel Cancer Screening Program.
