# Supplementary material for: Diversity, distribution, and population structure of Escherichia coli in the lower gastrointestinal tract of humans
Source: PLoS One. 2025 Jul 10;20(7):e0328147. doi: 10.1371/journal.pone.0328147 (PMC12244825; doi:10.1371/journal.pone.0328147)
Supplement: S2 Table — (DOCX) [file pone.0328147.s002.docx]

S2 Table. Individual’s demography, disease status, antibiotic consumption, sampled gut regions, retrieved total isolates and genotype(s).

| **Individual** | **Age** | **Sex** | **Disease** | **Antibiotic consumption within six months (at the time of colonoscopy)** | **Gut regions sampled** | **Retrieved *E coli* isolates** | ***E. coli* richness** |
| --- | --- | --- | --- | --- | --- | --- | --- |
| 1 | 64 | M | N | - | 2 | 62 | 1 |
| 3 | 63 | M | N | - | 3 | 98 | 6 |
| 4 | 63 | M | N | - | 2 | 50 | 6 |
| 5 | 61 | F | N | No | 3 | 88 | 3 |
| 7 | 70 | F | N | Yes | 3 | 40 | 5 |
| 8 | 59 | F | N | No | 3 | 69 | 1 |
| 9 | 67 | M | N | No | 3 | 93 | 4 |
| 11 | 52 | M | N | No | 3 | 93 | 2 |
| 12 | 64 | M | N | No | 3 | 93 | 1 |
| 13 | 68 | M | N | Yes | 3 | 93 | 1 |
| 14 | 51 | F | N | No | 3 | 93 | 3 |
| 15 | 85 | F | N | Yes | 2 | 62 | 1 |
| 16 | 36 | F | UC | No | 2 | 36 | 3 |
| 17 | 33 | M | N | No | 3 | 93 | 5 |
| 18 | 73 | F | N | No | 3 | 93 | 2 |
| 19 | 43 | M | N | No | 3 | 78 | 3 |
| 20 | 58 | F | N | No | 3 | 84 | 1 |
| 21 | 35 | F | CD | No | 3 | 93 | 2 |
| 22 | 62 | M | N | Yes | 3 | 85 | 6 |
| 23 | 54 | M | N | Yes | 3 | 54 | 4 |
| 24 | 54 | M | N | No | 3 | 88 | 2 |
| 25 | 62 | F | N | Yes | 3 | 92 | 1 |
| 26 | 57 | F | N | No | 3 | 50 | 1 |
| 27 | 28 | F | CD | Yes | 3 | 76 | 2 |
| 28 | 59 | F | N | No | 3 | 92 | 1 |
| 29 | 28 | F | N | Yes | 3 | 89 | 1 |
| 30 | 38 | F | N | No | 3 | 54 | 1 |
| 31 | 65 | F | N | No | 3 | 45 | 2 |
| 32 | 20 | F | N | No | 3 | 49 | 3 |
| 33 | 57 | M | CD | No | 3 | 84 | 1 |
| 34 | 55 | F | N | No | 3 | 91 | 5 |
| 35 | 78 | M | N | Yes | 3 | 43 | 3 |
| 36 | 71 | M | N | No | 3 | 45 | 2 |
| 37 | 69 | F | N | No | 3 | 87 | 3 |
| 38 | 30 | M | CD | Yes | 3 | 79 | 1 |
| 39 | 62 | M | N | No | 2 | 37 | 2 |
| 41 | 61 | F | N | No | 3 | 62 | 1 |
| 42 | 39 | M | N | Yes | 3 | 87 | 2 |
| 43 | 59 | M | N | No | 2 | 62 | 3 |
| 44 | 55 | M | N | No | 3 | 80 | 1 |
| 45 | 65 | F | N | No | 2 | 80 | 3 |
| 46 | 45 | M | N | Yes | 3 | 93 | 5 |
| 47 | 57 | F | N | No | 3 | 39 | 2 |
| 48 | 56 | M | N | Yes | 3 | 93 | 3 |
| 49 | 41 | F | N | No | 3 | 45 | 1 |
| 50 | 27 | F | N | Yes | 3 | 63 | 3 |

**Samples from individuals- 2, 6, 10 and 40 did not result in *E. coli* growth and were excluded from analysis; M: Male, F: Female; N: Individuals without Crohn’s disease (CD), Ulcerative colitis (UC) and Diarrheal conditions;** **“-”: data not available.**
