# Supplementary material for: Diversity, distribution, and population structure of Escherichia coli in the lower gastrointestinal tract of humans
Source: PLoS One. 2025 Jul 10;20(7):e0328147. doi: 10.1371/journal.pone.0328147 (PMC12244825; doi:10.1371/journal.pone.0328147)
Supplement: S3 Table — (DOCX) [file pone.0328147.s003.docx]

S3 Table. Frequency of unique strains isolated from respective gut regions and their classification into phylogroups, four major STs, B2 sub-types, and individual types based on strain distribution.

| Individual’s number | Gut region | Unique strain | Identified phylogroup | Identified ST by Doumith profiling | B2-subtyping | Frequency of the unique strain | Individual type |
| --- | --- | --- | --- | --- | --- | --- | --- |
| 1 | Colon | 1 | B2 | Nil | UA | 31 | Individual type A |
| 1 | Rectum | 1 | B2 | Nil | UA | 31 | Individual type A |
| 3 | Ileum | 1 | E | N/A | N/A | 1 | Individual type B and C |
| 3 | Ileum | 2 | B1 | N/A | N/A | 30 | Individual type B and C |
| 3 | Colon | 2 | B1 | N/A | N/A | 30 | Individual type B and C |
| 3 | Rectum | 2 | B1 | N/A | N/A | 31 | Individual type B and C |
| 3 | Colon | 3 | E | N/A | N/A | 1 | Individual type B and C |
| 3 | Ileum | 4 | B1 | N/A | N/A | 1 | Individual type B and C |
| 3 | Rectum | 4 | B1 | N/A | N/A | 2 | Individual type B and C |
| 3 | Rectum | 5 | D | Nil | N/A | 1 | Individual type B and C |
| 3 | Rectum | 6 | B1 | N/A | N/A | 1 | Individual type B and C |
| 4 | Colon | 1 | B2 | Nil | UA | 15 | Individual type B and C |
| 4 | Rectum | 1 | B2 | Nil | UA | 15 | Individual type B and C |
| 4 | Colon | 2 | F | N/A | N/A | 10 | Individual type B and C |
| 4 | Rectum | 2 | F | N/A | N/A | 2 | Individual type B and C |
| 4 | Colon | 3 | B2 | Nil | III (STc127) | 4 | Individual type B and C |
| 4 | Rectum | 3 | B2 | Nil | III (STc127) | 1 | Individual type B and C |
| 4 | Colon | 4 | F | N/A | N/A | 1 | Individual type B and C |
| 4 | Rectum | 5 | F | N/A | N/A | 1 | Individual type B and C |
| 4 | Rectum | 6 | F | N/A | N/A | 1 | Individual type B and C |
| 5 | Ileum | 1 | A | N/A | N/A | 1 | Extended version of individual type B and C or D and E |
| 5 | Ileum | 2 | A | N/A | N/A | 14 | Extended version of individual type B and C or D and E |
| 5 | Colon | 2 | A | N/A | N/A | 20 | Extended version of individual type B and C or D and E |
| 5 | Rectum | 2 | A | N/A | N/A | 12 | Extended version of individual type B and C or D and E |
| 5 | Ileum | 3 | B2 | 95 | IX (STc95) | 14 | Extended version of individual type B and C or D and E |
| 5 | Colon | 3 | B2 | 95 | IX (STc95) | 14 | Extended version of individual type B and C or D and E |
| 5 | Rectum | 3 | B2 | 95 | IX (STc95) | 13 | Extended version of individual type B and C or D and E |
| 7 | Ileum | 1 | B2 | 131 | I (STc131) | 2 | Individual type D and E |
| 7 | Colon | 1 | B2 | 131 | I (STc131) | 2 | Individual type D and E |
| 7 | Ileum | 2 | B2 | 131 | I (STc131) | 3 | Individual type D and E |
| 7 | Colon | 2 | B2 | 131 | I (STc131) | 5 | Individual type D and E |
| 7 | Rectum | 2 | B2 | 131 | I (STc131) | 14 | Individual type D and E |
| 7 | Ileum | 3 | B1 | N/A | N/A | 6 | Individual type D and E |
| 7 | Colon | 3 | B1 | N/A | N/A | 3 | Individual type D and E |
| 7 | Rectum | 3 | B1 | N/A | N/A | 1 | Individual type D and E |
| 7 | Ileum | 4 | B1 | N/A | N/A | 2 | Individual type D and E |
| 7 | Rectum | 5 | B1 | N/A | N/A | 1 | Individual type D and E |
| 7 | Colon | 5 | B1 | N/A | N/A | 1 | Individual type D and E |
| 8 | Ileum | 1 | B2 | 73 | II (STc73) | 26 | Individual type A |
| 8 | Colon | 1 | B2 | 73 | II (STc73) | 16 | Individual type A |
| 8 | Rectum | 1 | B2 | 73 | II (STc73) | 27 | Individual type A |
| 9 | Ileum | 1 | B2 | 131 | I (STc131) | 28 | Individual type B and C |
| 9 | Colon | 1 | B2 | 131 | I (STc131) | 28 | Individual type B and C |
| 9 | Rectum | 1 | B2 | 131 | I (STc131) | 26 | Individual type B and C |
| 9 | Ileum | 2 | B2 | 95 | IX (STc95) | 3 | Individual type B and C |
| 9 | Colon | 2 | B2 | 95 | IX (STc95) | 2 | Individual type B and C |
| 9 | Rectum | 2 | B2 | 95 | IX (STc95) | 3 | Individual type B and C |
| 9 | Colon | 3 | B2 | 95 | IX (STc95) | 1 | Individual type B and C |
| 9 | Rectum | 4 | B2 | 95 | IX (STc95) | 2 | Individual type B and C |
| 11 | Ileum | 1 | F | N/A | N/A | 25 | Extended version of individual type B and C or D and E |
| 11 | Colon | 1 | F | N/A | N/A | 16 | Extended version of individual type B and C or D and E |
| 11 | Rectum | 1 | F | N/A | N/A | 16 | Extended version of individual type B and C or D and E |
| 11 | Ileum | 2 | B2 | 73 | II (STc73) | 6 | Extended version of individual type B and C or D and E |
| 11 | Colon | 2 | B2 | 73 | II (STc73) | 15 | Extended version of individual type B and C or D and E |
| 11 | Rectum | 2 | B2 | 73 | II (STc73) | 15 | Extended version of individual type B and C or D and E |
| 12 | Ileum | 1 | B2 | 131 | I (STc131) | 31 | Individual type A |
| 12 | Colon | 1 | B2 | 131 | I (STc131) | 31 | Individual type A |
| 12 | Rectum | 1 | B2 | 131 | I (STc131) | 31 | Individual type A |
| 13 | Ileum | 1 | B2 | 131 | I (STc131) | 31 | Individual type A |
| 13 | Colon | 1 | B2 | 131 | I (STc131) | 31 | Individual type A |
| 13 | Rectum | 1 | B2 | 131 | I (STc131) | 31 | Individual type A |
| 14 | Ileum | 1 | B2 | Nil | UA | 23 | Individual type B and C |
| 14 | Colon | 1 | B2 | Nil | UA | 20 | Individual type B and C |
| 14 | Rectum | 1 | B2 | Nil | UA | 31 | Individual type B and C |
| 14 | Ileum | 2 | A | N/A | N/A | 8 | Individual type B and C |
| 14 | Colon | 2 | A | N/A | N/A | 7 | Individual type B and C |
| 14 | Colon | 3 | B2 | Nil | II (STc73) | 4 | Individual type B and C |
| 15 | Colon | 1 | E | N/A | N/A | 31 | Individual type A |
| 15 | Rectum | 1 | E | N/A | N/A | 31 | Individual type A |
| 16 | Ileum | 1 | B2 | Nil | VI (STc12) | 13 | Individual type B and C |
| 16 | Rectum | 1 | B2 | Nil | VI (STc12) | 13 | Individual type B and C |
| 16 | Ileum | 2 | D | 69 | N/A | 8 | Individual type B and C |
| 16 | Rectum | 2 | D | 69 | N/A | 1 | Individual type B and C |
| 16 | Rectum | 3 | A | N/A | N/A | 1 | Individual type B and C |
| 17 | Ileum | 1 | B2 | 95 | IX (STc95) | 12 | Extended version of individual type B and C or D and E |
| 17 | Colon | 1 | B2 | 95 | IX (STc95) | 11 | Extended version of individual type B and C or D and E |
| 17 | Rectum | 1 | B2 | 95 | IX (STc95) | 15 | Extended version of individual type B and C or D and E |
| 17 | Ileum | 2 | E | N/A | N/A | 11 | Extended version of individual type B and C or D and E |
| 17 | Colon | 2 | E | N/A | N/A | 3 | Extended version of individual type B and C or D and E |
| 17 | Ileum | 3 | A | N/A | N/A | 8 | Extended version of individual type B and C or D and E |
| 17 | Colon | 3 | A | N/A | N/A | 15 | Extended version of individual type B and C or D and E |
| 17 | Rectum | 3 | A | N/A | N/A | 12 | Extended version of individual type B and C or D and E |
| 17 | Colon | 4 | B2 | 95 | IX (STc95) | 2 | Extended version of individual type B and C or D and E |
| 17 | Rectum | 5 | A | N/A | N/A | 4 | Extended version of individual type B and C or D and E |
| 18 | Ileum | 1 | A | N/A | N/A | 31 | Individual type F |
| 18 | Colon | 1 | A | N/A | N/A | 31 | Individual type F |
| 18 | Rectum | 2 | A | N/A | N/A | 31 | Individual type F |
| 19 | Ileum | 1 | B2 | Nil | UA | 29 | Individual type B and C |
| 19 | Colon | 1 | B2 | Nil | UA | 22 | Individual type B and C |
| 19 | Rectum | 1 | B2 | Nil | UA | 24 | Individual type B and C |
| 19 | Ileum | 2 | B2 | Nil | IV (STc141) | 1 | Individual type B and C |
| 19 | Colon | 3 | B2 | Nil | IV (STc141) | 2 | Individual type B and C |
| 20 | Ileum | 1 | D | Nil | N/A | 31 | Individual type A |
| 20 | Colon | 1 | D | Nil | N/A | 22 | Individual type A |
| 20 | Rectum | 1 | D | Nil | N/A | 31 | Individual type A |
| 21 | Ileum | 1 | B2 | 95 | IX (STc95) | 27 | Individual type D and E |
| 21 | Colon | 1 | B2 | 95 | IX (STc95) | 29 | Individual type D and E |
| 21 | Rectum | 1 | B2 | 95 | IX (STc95) | 1 | Individual type D and E |
| 21 | Ileum | 2 | B2 | 73 | II (STc73) | 4 | Individual type D and E |
| 21 | Colon | 2 | B2 | 73 | II (STc73) | 2 | Individual type D and E |
| 21 | Rectum | 2 | B2 | 73 | II (STc73) | 30 | Individual type D and E |
| 22 | Ileum | 1 | A | N/A | N/A | 22 | Individual type B and C |
| 22 | Colon | 1 | A | N/A | N/A | 22 | Individual type B and C |
| 22 | Rectum | 1 | A | N/A | N/A | 20 | Individual type B and C |
| 22 | Ileum | 2 | B1 | N/A | N/A | 1 | Individual type B and C |
| 22 | Rectum | 2 | B1 | N/A | N/A | 2 | Individual type B and C |
| 22 | Ileum | 3 | D | Nil | N/A | 5 | Individual type B and C |
| 22 | Colon | 3 | D | Nil | N/A | 4 | Individual type B and C |
| 22 | Rectum | 3 | D | Nil | N/A | 4 | Individual type B and C |
| 22 | Ileum | 4 | *E*. clade I | N/A | N/A | 1 | Individual type B and C |
| 22 | Rectum | 5 | *E*. clade I | N/A | N/A | 1 | Individual type B and C |
| 22 | Colon | 6 | B2 | 95 | IX (STc95) | 2 | Individual type B and C |
| 22 | Rectum | 6 | B2 | 95 | IX (STc95) | 1 | Individual type B and C |
| 23 | Ileum | 1 | D | 69 | N/A | 3 | Individual type D and E |
| 23 | Colon | 1 | D | 69 | N/A | 4 | Individual type D and E |
| 23 | Rectum | 1 | D | 69 | N/A | 5 | Individual type D and E |
| 23 | Ileum | 2 | A | N/A | N/A | 14 | Individual type D and E |
| 23 | Colon | 2 | A | N/A | N/A | 8 | Individual type D and E |
| 23 | Rectum | 2 | A | N/A | N/A | 14 | Individual type D and E |
| 23 | Ileum | 3 | B2 | 131 | I (STc131) | 1 | Individual type D and E |
| 23 | Colon | 3 | B2 | 131 | I (STc131) | 2 | Individual type D and E |
| 23 | Rectum | 3 | B2 | 131 | I (STc131) | 1 | Individual type D and E |
| 23 | Rectum | 4 | A | N/A | N/A | 2 | Individual type D and E |
| 24 | Ileum | 1 | B2 | 95 | UA | 29 | Individual type B and C |
| 24 | Colon | 1 | B2 | 95 | UA | 26 | Individual type B and C |
| 24 | Rectum | 1 | B2 | 95 | UA | 31 | Individual type B and C |
| 24 | Ileum | 2 | B2 | Nil | IV (STc141) | 1 | Individual type B and C |
| 24 | Colon | 2 | B2 | Nil | IV (STc141) | 1 | Individual type B and C |
| 25 | Ileum | 1 | B2 | 95 | IX (STc95) | 31 | Individual type A |
| 25 | Colon | 1 | B2 | 95 | IX (STc95) | 31 | Individual type A |
| 25 | Rectum | 1 | B2 | 95 | IX (STc95) | 30 | Individual type A |
| 26 | Ileum | 1 | D | Nil | N/A | 14 | Individual type A |
| 26 | Colon | 1 | D | Nil | N/A | 18 | Individual type A |
| 26 | Rectum | 1 | D | Nil | N/A | 18 | Individual type A |
| 27 | Ileum | 1 | B2 | Nil | X (STc372) | 24 | Individual type B and C |
| 27 | Colon | 1 | B2 | Nil | X (STc372) | 27 | Individual type B and C |
| 27 | Rectum | 1 | B2 | Nil | X (STc372) | 24 | Individual type B and C |
| 27 | Ileum | 2 | B2 | * | * | 1 | Individual type B and C |
| 28 | Ileum | 1 | B1 | N/A | N/A | 31 | Individual type A |
| 28 | Colon | 1 | B1 | N/A | N/A | 31 | Individual type A |
| 28 | Rectum | 1 | B1 | N/A | N/A | 30 | Individual type A |
| 29 | Ileum | 1 | B2 | 131 | I (STc131) | 31 | Individual type A |
| 29 | Colon | 1 | B2 | 131 | I (STc131) | 28 | Individual type A |
| 29 | Rectum | 1 | B2 | 131 | I (STc131) | 30 | Individual type A |
| 30 | Ileum | 1 | B2 | 95 | IX (STc95) | 11 | Individual type A |
| 30 | Colon | 1 | B2 | 95 | IX (STc95) | 22 | Individual type A |
| 30 | Rectum | 1 | B2 | 95 | IX (STc95) | 21 | Individual type A |
| 31 | Ileum | 1 | B2 | Nil | IV (STc141) | 1 | Individual type B and C |
| 31 | Colon | 1 | B2 | Nil | IV (STc141) | 4 | Individual type B and C |
| 31 | Ileum | 2 | B2 | 131 | I (STc131) | 22 | Individual type B and C |
| 31 | Colon | 2 | B2 | 131 | I (STc131) | 18 | Individual type B and C |
| 32 | Ileum | 1 | B2 | Nil | VI (STc12) | 20 | Individual type B and C |
| 32 | Colon | 1 | B2 | Nil | VI (STc12) | 13 | Individual type B and C |
| 32 | Rectum | 1 | B2 | Nil | VI (STc12) | 14 | Individual type B and C |
| 32 | Colon | 2 | B2 | Nil | VI (STc12) | 1 | Individual type B and C |
| 32 | Rectum | 3 | B1 | N/A | N/A | 1 | Individual type B and C |
| 33 | Ileum | 1 | B2 | Nil | UA | 26 | Individual type A |
| 33 | Colon | 1 | B2 | Nil | UA | 29 | Individual type A |
| 33 | Rectum | 1 | B2 | Nil | UA | 29 | Individual type A |
| 34 | Ileum | 1 | A | N/A | N/A | 16 | Extended version of individual type B and C or D and E |
| 34 | Colon | 1 | A | N/A | N/A | 10 | Extended version of individual type B and C or D and E |
| 34 | Rectum | 1 | A | N/A | N/A | 9 | Extended version of individual type B and C or D and E |
| 34 | Ileum | 2 | F | N/A | N/A | 15 | Extended version of individual type B and C or D and E |
| 34 | Colon | 2 | F | N/A | N/A | 14 | Extended version of individual type B and C or D and E |
| 34 | Rectum | 2 | F | N/A | N/A | 16 | Extended version of individual type B and C or D and E |
| 34 | Colon | 3 | A | N/A | N/A | 5 | Extended version of individual type B and C or D and E |
| 34 | Rectum | 3 | A | N/A | N/A | 3 | Extended version of individual type B and C or D and E |
| 34 | Colon | 4 | A | N/A | N/A | 2 | Extended version of individual type B and C or D and E |
| 34 | Rectum | 5 | B2 | Nil | VI (STc12) | 1 | Extended version of individual type B and C or D and E |
| 35 | Ileum | 1 | B1 | N/A | N/A | 9 | Individual type B and C |
| 35 | Colon | 1 | B1 | N/A | N/A | 15 | Individual type B and C |
| 35 | Rectum | 1 | B1 | N/A | N/A | 11 | Individual type B and C |
| 35 | Ileum | 2 | B1 | N/A | N/A | 2 | Individual type B and C |
| 35 | Ileum | 3 | B2 | 95 | IX (STc95) | 1 | Individual type B and C |
| 35 | Rectum | 3 | B2 | 95 | IX (STc95) | 5 | Individual type B and C |
| 36 | Ileum | 1 | B2 | Nil | VI (STc12) | 15 | Individual type B and C |
| 36 | Colon | 1 | B2 | Nil | VI (STc12) | 13 | Individual type B and C |
| 36 | Rectum | 1 | B2 | Nil | VI (STc12) | 13 | Individual type B and C |
| 36 | Colon | 2 | A | N/A | N/A | 1 | Individual type B and C |
| 36 | Rectum | 2 | A | N/A | N/A | 3 | Individual type B and C |
| 37 | Ileum | 1 | A | N/A | N/A | 26 | Individual type B and C |
| 37 | Colon | 1 | A | N/A | N/A | 29 | Individual type B and C |
| 37 | Rectum | 1 | A | N/A | N/A | 28 | Individual type B and C |
| 37 | Ileum | 2 | D | Nil | N/A | 1 | Individual type B and C |
| 37 | Colon | 2 | D | Nil | N/A | 1 | Individual type B and C |
| 37 | Ileum | 3 | A | N/A | N/A | 1 | Individual type B and C |
| 37 | Colon | 3 | A | N/A | N/A | 1 | Individual type B and C |
| 38 | Ileum | 1 | D | Nil | N/A | 26 | Individual type A |
| 38 | Colon | 1 | D | Nil | N/A | 27 | Individual type A |
| 38 | Rectum | 1 | D | Nil | N/A | 26 | Individual type A |
| 39 | Colon | 1 | B2 | 73 | II (STc73) | 18 | Individual type B and C |
| 39 | Rectum | 1 | B2 | 73 | II (STc73) | 17 | Individual type B and C |
| 39 | Colon | 2 | A | N/A | N/A | 1 | Individual type B and C |
| 39 | Rectum | 2 | A | N/A | N/A | 1 | Individual type B and C |
| 41 | Ileum | 1 | A | N/A | N/A | 31 | Individual type A |
| 41 | Colon | 1 | A | N/A | N/A | 31 | Individual type A |
| 42 | Ileum | 1 | B2 | 131 | UA | 27 | Individual type B and C |
| 42 | Colon | 1 | B2 | 131 | UA | 24 | Individual type B and C |
| 42 | Rectum | 1 | B2 | 131 | UA | 30 | Individual type B and C |
| 42 | Ileum | 2 | B2 | 95 | IX (STc95) | 2 | Individual type B and C |
| 42 | Colon | 2 | B2 | 95 | IX (STc95) | 3 | Individual type B and C |
| 42 | Rectum | 2 | B2 | 95 | IX (STc95) | 1 | Individual type B and C |
| 43 | Colon | 1 | A | N/A | N/A | 16 | Extended version of individual type B and C or D and E |
| 43 | Rectum | 1 | A | N/A | N/A | 13 | Extended version of individual type B and C or D and E |
| 43 | Colon | 2 | A | N/A | N/A | 13 | Extended version of individual type B and C or D and E |
| 43 | Rectum | 2 | A | N/A | N/A | 12 | Extended version of individual type B and C or D and E |
| 43 | Colon | 3 | D | Nil | N/A | 2 | Extended version of individual type B and C or D and E |
| 43 | Rectum | 3 | D | Nil | N/A | 6 | Extended version of individual type B and C or D and E |
| 44 | Ileum | 1 | B2 | Nil | UA | 30 | Individual type A |
| 44 | Colon | 1 | B2 | Nil | UA | 27 | Individual type A |
| 44 | Rectum | 1 | B2 | Nil | UA | 23 | Individual type A |
| 45 | Ileum | 1 | B2 | Nil | X (STc372) | 18 | Extended version of individual type B and C or D and E |
| 45 | Colon | 1 | B2 | Nil | X (STc372) | 18 | Extended version of individual type B and C or D and E |
| 45 | Rectum | 1 | B2 | Nil | X (STc372) | 1 | Extended version of individual type B and C or D and E |
| 45 | Colon | 2 | B2 | Nil | IX (STc95) | 12 | Extended version of individual type B and C or D and E |
| 45 | Rectum | 2 | B2 | Nil | IX (STc95) | 26 | Extended version of individual type B and C or D and E |
| 45 | Colon | 3 | B2 | Nil | IX (STc95) | 1 | Extended version of individual type B and C or D and E |
| 45 | Rectum | 3 | B2 | Nil | IX (STc95) | 4 | Extended version of individual type B and C or D and E |
| 46 | Ileum | 1 | D | Nil | N/A | 24 | Individual type B and C |
| 46 | Colon | 1 | D | Nil | N/A | 22 | Individual type B and C |
| 46 | Rectum | 1 | D | Nil | N/A | 25 | Individual type B and C |
| 46 | Ileum | 2 | B1 | N/A | N/A | 1 | Individual type B and C |
| 46 | Colon | 2 | B1 | N/A | N/A | 7 | Individual type B and C |
| 46 | Rectum | 2 | B1 | N/A | N/A | 5 | Individual type B and C |
| 46 | Ileum | 3 | B1 | N/A | N/A | 1 | Individual type B and C |
| 46 | Ileum | 4 | A | N/A | N/A | 4 | Individual type B and C |
| 46 | Rectum | 4 | A | N/A | N/A | 1 | Individual type B and C |
| 46 | Ileum | 5 | D | Nil | N/A | 1 | Individual type B and C |
| 46 | Colon | 5 | D | Nil | N/A | 2 | Individual type B and C |
| 47 | Ileum | 1 | B2 | Nil | UA | 13 | Individual type D and E |
| 47 | Colon | 1 | B2 | Nil | UA | 19 | Individual type D and E |
| 47 | Rectum | 1 | B2 | Nil | UA | 2 | Individual type D and E |
| 47 | Ileum | 2 | F | N/A | N/A | 3 | Individual type D and E |
| 47 | Colon | 2 | F | N/A | N/A | 1 | Individual type D and E |
| 47 | Rectum | 2 | F | N/A | N/A | 1 | Individual type D and E |
| 48 | Ileum | 1 | B1 | N/A | N/A | 8 | Individual type B and C |
| 48 | Colon | 1 | B1 | N/A | N/A | 12 | Individual type B and C |
| 48 | Rectum | 1 | B1 | N/A | N/A | 7 | Individual type B and C |
| 48 | Ileum | 2 | B2 | Nil | VII (STc14) | 23 | Individual type B and C |
| 48 | Colon | 2 | B2 | Nil | VII (STc14) | 18 | Individual type B and C |
| 48 | Rectum | 2 | B2 | Nil | VII (STc14) | 22 | Individual type B and C |
| 48 | Colon | 3 | A | N/A | N/A | 1 | Individual type B and C |
| 48 | Rectum | 3 | A | N/A | N/A | 2 | Individual type B and C |
| 49 | Ileum | 1 | D | Nil | N/A | 21 | Individual type A |
| 49 | Colon | 1 | D | Nil | N/A | 24 | Individual type A |
| 50 | Ileum | 1 | B2 | Nil | VII (STc14) | 20 | Individual type D and E |
| 50 | Colon | 1 | B2 | Nil | VII (STc14) | 8 | Individual type D and E |
| 50 | Rectum | 1 | B2 | Nil | VII (STc14) | 21 | Individual type D and E |
| 50 | Ileum | 2 | B1 | N/A | N/A | 8 | Individual type D and E |
| 50 | Rectum | 2 | B1 | N/A | N/A | 4 | Individual type D and E |
| 50 | Rectum | 3 | D | Nil | N/A | 2 | Individual type D and E |

‘Nil’: Indicates that no band was found after the gel electrophoresis, to assign the respective B2 and D isolates as ST73, ST95, ST131 and ST69, respectively, according to Doumith profiling.; ‘*’: Despite multiple gel electrophoresis runs, the strain failed to show band at a later stage.
