# Supplementary material for: Diversity, distribution, and population structure of Escherichia coli in the lower gastrointestinal tract of humans
Source: PLoS One. 2025 Jul 10;20(7):e0328147. doi: 10.1371/journal.pone.0328147 (PMC12244825; doi:10.1371/journal.pone.0328147)
Supplement: S4 Table — (DOCX) [file pone.0328147.s004.docx]

S4 Table. Cell χ2 value showing the relationship between the phylogroup of the most and the second most abundant strains in an individual.

| Phylogroup of second most dominant strain | | | | | | |
| --- | --- | --- | --- | --- | --- | --- |
|  |  |  | A | B1 | B2 | D |
| Phylogroup of most dominant strain | A | Observed | 4 | 0 | 1 | 2 |
|  |  | Observed-Expected | 2 | -1.25 | -2 | 1.25 |
|  |  | Cell χ2 | **2** | 1.25 | 1.33 | **2.08** |
|  | B1 | Observed | 0 | 1 | 1 | 0 |
|  |  | Observed-Expected | -0.57 | 0.64 | 0.14 | -0.21 |
|  |  | Cell χ2 | 0.57 | **1.16** | **0.02** | 0.21 |
|  | B2 | Observed | 4 | 3 | 10 | 1 |
|  |  | Observed-Expected | -1.14 | -0.21 | 2.29 | -0.93 |
|  |  | Cell χ2 | 0.25 | 0.01 | **0.68** | 0.45 |
|  | D | Observed | 0 | 1 | 0 | 0 |
|  |  | Observed-Expected | -0.29 | 0.82 | -0.43 | -0.11 |
|  |  | Cell χ2 | 0.29 | **3.78** | 0.43 | 0.11 |

Bold and light blue colour shows where the observed value was found greater than the expected value.
