# Supplementary material for: Diversity, distribution, and population structure of Escherichia coli in the lower gastrointestinal tract of humans
Source: PLoS One. 2025 Jul 10;20(7):e0328147. doi: 10.1371/journal.pone.0328147 (PMC12244825; doi:10.1371/journal.pone.0328147)
Supplement: S5 Table — (DOCX) [file pone.0328147.s005.docx]

S5 Table. A comparison between the current study and the study by Gordon *et al*. (2015) regarding the percentage of individuals carrying different number of unique strains.

| Number of unique strain(s) | Current study: Individuals (%) carrying unique strain(s) | Gordon *et al*. (2015) [1] study: Individuals (%) carrying unique strain(s) |
| --- | --- | --- |
| 1 | 35 (34.78) | 15 |
| 2 | 22 (21.74) | 25 |
| 3 | 24 (23.91) | 21 |
| 4 | 4 (4.35) | 14 |
| 5 | 9 (8.70) | 11 |
| 6 | 7 (6.52) | 6 |
| 7 | 0 | 6 |
| 8 | 0 | 2 |

Reference

1. Gordon DM, O'Brien CL, Pavli P. Escherichia coli diversity in the lower intestinal tract of humans. Environmental Microbiology Reports. 2015;7(4):642-8. doi: 10.1111/1758-2229.12300.
