# Supplementary figures and images for: Diversity, distribution, and population structure of Escherichia coli in the lower gastrointestinal tract of humans
Source: PLoS One. 2025 Jul 10;20(7):e0328147. doi: 10.1371/journal.pone.0328147 (PMC12244825; doi:10.1371/journal.pone.0328147)

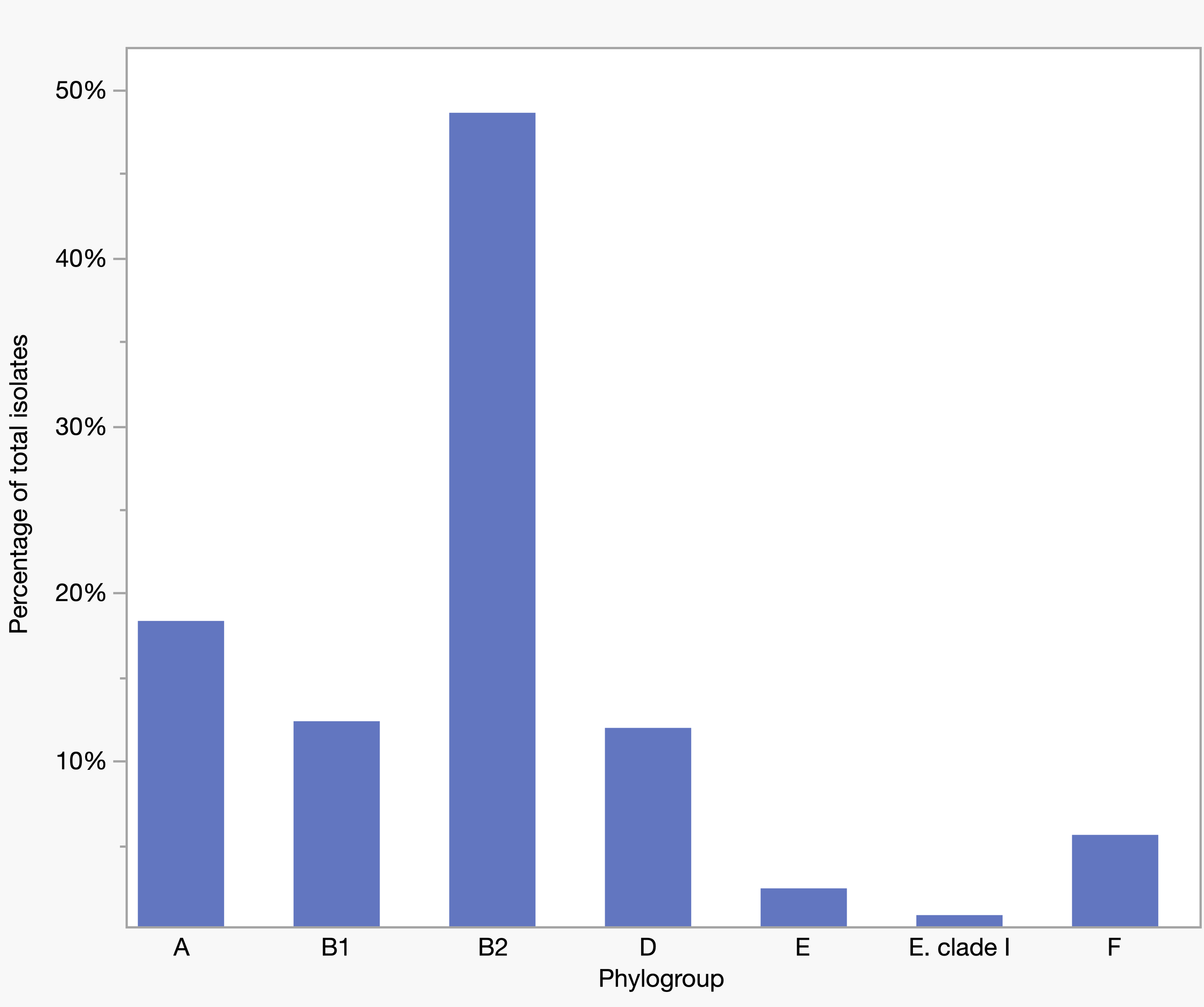

Supplement: S1 Fig — For example, a total of 251 unique strains were detected across different gut regions of 46 individuals, of which 122 were identified as phylogroup B2 strains, accounting for approximately 49% of the total. (TIF) [file pone.0328147.s006.tif]

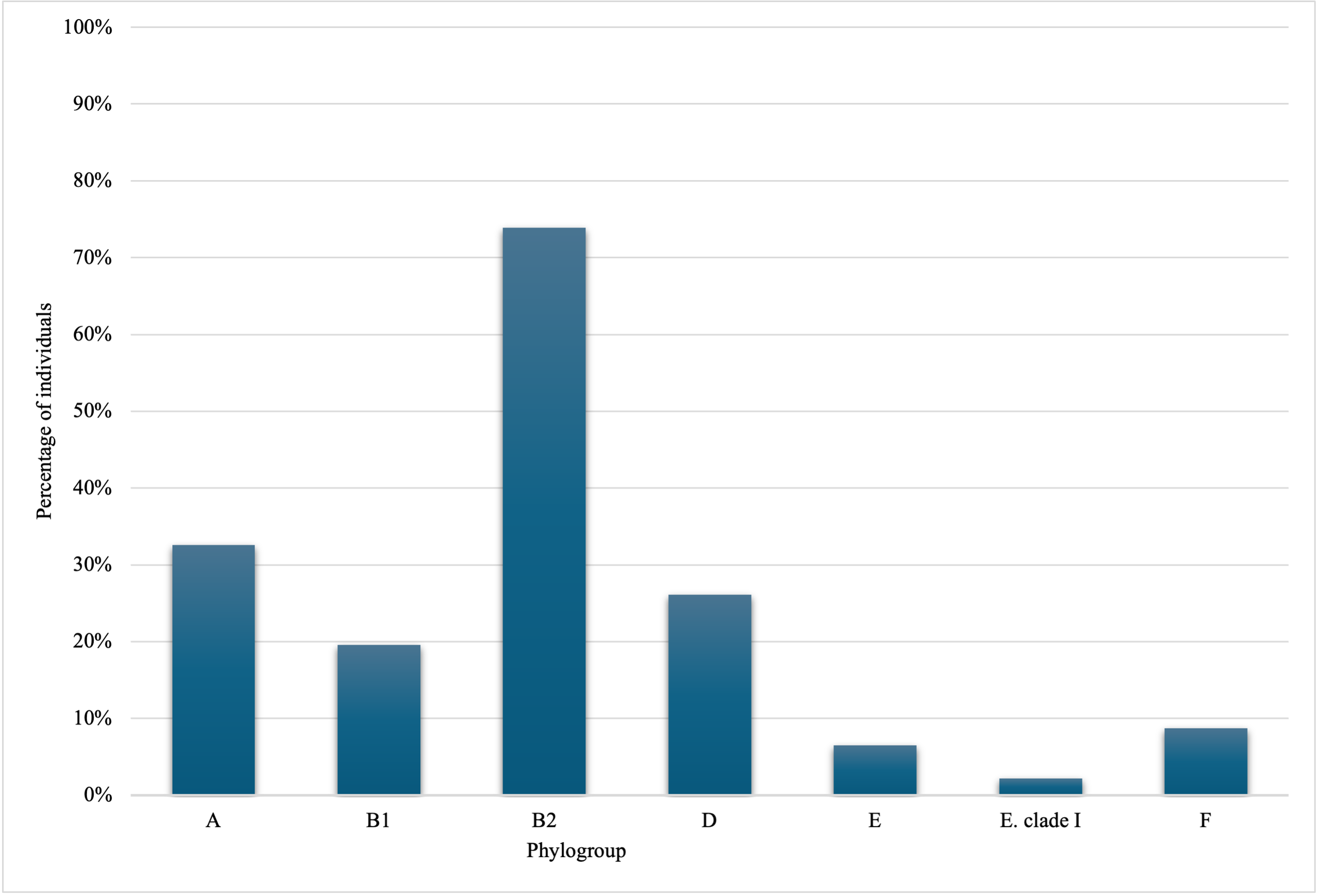

Supplement: S2 Fig — A total of 46 individuals were studied. For example, phylogroup B2 strains were retrieved from 34 individuals, accounting for approximately 74% (73.91%) of the total individuals. (TIF) [file pone.0328147.s007.tif]

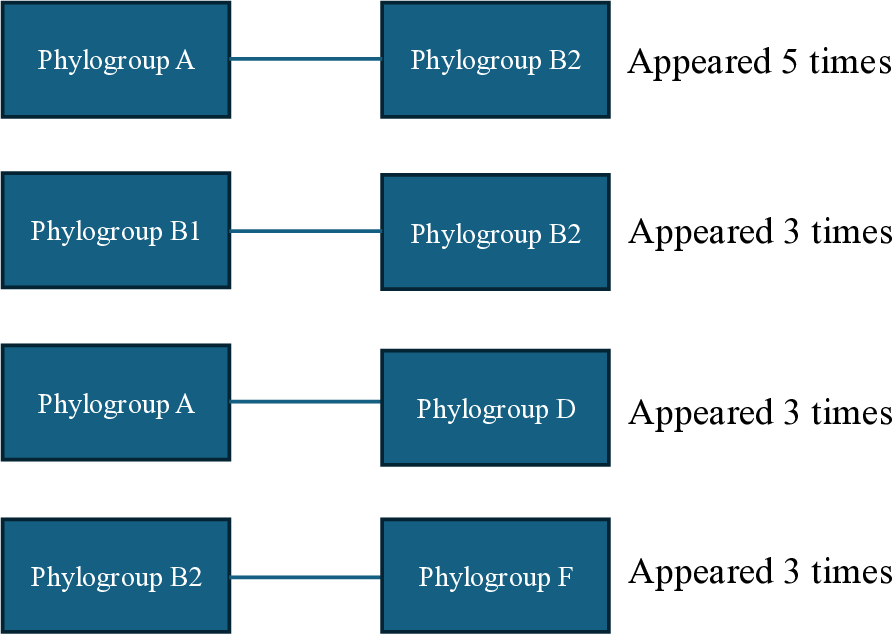

Supplement: S3 Fig — The combinations were found restricted to A-B2, B1-B2, A-D, and B2-F. (TIF) [file pone.0328147.s008.tif]
