# Supplementary material for: Diversity, distribution, and population structure of Escherichia coli in the lower gastrointestinal tract of humans
Source: PLoS One. 2025 Jul 10;20(7):e0328147. doi: 10.1371/journal.pone.0328147 (PMC12244825; doi:10.1371/journal.pone.0328147)
Supplement: Attachment — Submitted filename: Response to Reviewers.pdf [file pone.0328147.s011.pdf]

PONE-D-24-33067R2

Authors' response to the journal requirements and reviewers' comments are provided below for PONE-D-24-33067R2.

We have listed and explained all the changes below, so the revisions are easy to follow. This document includes two tables: 1) our response to the journal requirements, and 2) our response to each of the reviewer's comments.

1) Authors' response to journal requirements

Journal requirements	Authors' response
1. Please ensure that your manuscript meets PLoS ONE's style requirements, including those for file naming. The PLoS ONE style templates can be found at https://journals.plos.org/plosone/s/file?id=wjVg/PLoSOne_formatting_sample_main_body.pdf and https://journals.plos.org/plosone/s/file?id=ba62/PLoSOne_formatting_sample_title_authors_affiliations.pdf	**Authors:** We have followed PLoS ONE's style and requirements in preparing our revised manuscript.
2. Please include your full ethics statement in the 'Methods' section of your manuscript file. In your statement, please include the full name of the IRB or ethics committee who approved or waived your study, as well as whether or not you obtained informed written or verbal consent. If consent was waived for your study, please include this information in your statement as well.	**Authors:** Apologies for the oversight, we have now included more details about the ethical committee. The changes have been included in the 'Materials and methods' section under the heading 'Ethics statement' (Lines: 99-104).
3. Please upload a new copy of S1-S4 Figures as the detail is not clear. Please follow the link for more information: https://blogs.plos.org/plos/2019/06/looking-good-tips-for-creating-your-plos-figures-graphics/"" https://blogs.plos.org/plos/2019/06/looking-good-tips-for-creating-your-plos-figures-graphics/	**Authors:** We have revised S1-S3 Figures for better clarity, and the previous S4 Figure has now been presented as S5 Table. In addition, more information has been added to the legends of S1 and S2 Figures.
Please review your reference list to ensure that it is complete and correct. If you have cited papers that have been retracted, please include the rationale for doing so in the manuscript text, or remove these references and replace them with relevant current references. Any changes to the reference list should be mentioned in the rebuttal letter that accompanies your revised manuscript. If you need to cite a retracted article, indicate the	**Authors:** The manuscript does not contain any retracted papers in the reference list. Some citations (citations no. 17, 18, 19, and 21) have been newly included in the manuscript and reference list, as Reviewer #3 suggested adding the missing citation for literature mentioned in lines: 75-76.

article's retracted status in the References list and also include a citation and full reference for the retraction notice.	

2) Authors' response to reviewers' comments

Reviewer's response to questions		Authors' response
1. If the authors have adequately addressed your comments raised in a previous round of review and you feel that this manuscript is now acceptable for publication, you may indicate that here to bypass the "Comments to the Author" section, enter your conflict of interest statement in the "Confidential to Editor" section, and submit your "Accept" recommendation	Reviewer #3: (No Response)	-
	Reviewer #4: (No Response)	-
2. Is the manuscript technically sound, and do the data support the conclusions?<br><br>The manuscript must describe a technically sound piece of scientific research with data that supports the conclusions. Experiments must have been conducted rigorously, with appropriate controls, replication, and sample sizes. The conclusions must be drawn appropriately based on the data presented.	Reviewer #3: Yes	**Authors:** Thank you.
	Reviewer #4: Partly	**Authors:** Thank you. We would like to highlight the rigorous approach adopted in our study.<br>From 46 individuals (originally 50), we analysed a total of 3355 *E. coli* isolates, which represents a relatively large collection of fresh isolates from individuals. For molecular typing of *E. coli* isolates, we used a rapid, simple, and highly discriminatory MLVA PCR method. We maintained high standards during the laboratory work and analysed our data rigorously.<br>We were cautious not to overstate our findings and drew conclusions based on our observations, such as the higher abundance of B2 strains in all gut locations, the

		lack of variation of phylogroups or the four major STs across gut locations, and the identification of individual types based on strain distribution, and among others. Since our aim was to determine the distribution and diversity of *E. coli* population structure in the lower gut, this study enabled us to examine both population and sub-population structures of *E. coli* across different gut locations of the lower gut. Observing location-specific distribution of strains would not have been possible by analysing faecal samples alone, but our study made this possible by using gut biopsy specimens instead of faecal specimens.
3. Has the statistical analysis been performed appropriately and rigorously?	Reviewer #3: Yes	**Authors:** Thank you.
	Reviewer #4: I Don't Know	**Authors:** Thank you. Actually, the manuscript includes a subsection titled ‘Statistical analysis’ under the ‘Materials and methods’ section (Lines: 163-170).<br><br>However, we would like to kindly point out where the statistical analyses were carried out in our study. We used JMP software for statistical analysis of our data and visualization of most of the graphs. We performed statistical analysis of our data wherever applicable, such as analysing unique strains per individual; frequency of unique strains according to gender, age, sex, disease status; distribution of four phylogroups in individuals for antibiotic use; strain richness and diversity; phylogroup and four major sequence types of

		variation according to gut locations, unique strain heterogeneity, etc.
4. Have the authors made all data underlying the findings in their manuscript fully available?<br><br>The PLOS Data policy requires authors to make all data underlying the findings described in their manuscript fully available without restriction, with rare exception (please refer to the Data Availability Statement in the manuscript PDF file). The data should be provided as part of the manuscript or its supporting information or deposited to a public repository. For example, in addition to summary statistics, the data points behind means, medians and variance measures should be available. If there are restrictions on publicly sharing data—e.g. participant privacy or use of data from a third party—those must be specified.	Reviewer #3: Yes	**Authors:** Thank you.
	Reviewer #4: No	**Authors:** Thank you. We have included our raw data, including the metadata in the supplementary section. For example, Table S1 contains information on individuals number, indication for colonoscopy, and ongoing medication; Table S2 provides information on individuals' age, sex, disease condition, antibiotic consumption, number of gut regions studied per sample, retrieval of isolates and strain richness; Table S3 includes the data on frequency of unique strains isolated from respective gut regions and their classification into phylogroups, four major STs, B2 sub-types, and individual types based on strain distribution.
5. Is the manuscript presented in an intelligible fashion and written in standard English?<br><br>PLoS ONE does not copyedit accepted manuscripts, so the language in submitted articles must be clear, correct, and unambiguous. Any typographical or grammatical errors should be corrected at revision, so please note any specific errors here.	Reviewer #3: Yes<br>Reviewer #4: Yes	**Authors:** Thank you.

6. Review Comments by reviewer and authors' responses.	
**Reviewer #3:** This paper was well written, and methods and experiments explained in detail. I have minor comments that are listed below.	**Authors:** We appreciate this assessment and have revised the manuscript according to the reviewer's comments/ suggestions, which are detailed below.
Introduction<br>Line 70-71: missing reference "The main niche for E. coli in humans is the lower region of the gut: the ileum, colon, and rectum [ ]"	Introduction<br>Line 75-76: We have provided references for the information and slightly paraphrased the sentences.
Line 72-73: in the sentence "The isolation of E. coli strains and their characteristics may vary from region to region in the intestinal tract of pigs [20].", the mentioning of pigs came out of nowhere. Can you rephrase so the transition be smoother between human and pig research	Line 78-81: As advised, the sentence has been paraphrased. The example of the pig study has been categorized under mammals, which would provide a smoother transition from pig research to human research.
Materials and methods<br>3) Line 147: having E clade twice "…A, B1, B2, D, E, F, and E clade." Why are clade C, G and H not included?<br>4) Line 148: having E clade twice "…or E and E clade."	Materials and methods<br>We apologise for the confusion- we have corrected the text and now provided the full form, where E refers to phylogroup E, and E clade refers to the *Escherichia* clades (please refer to lines 157 and 158). For phylogrouping of our *E. coli* strains, we followed the highly cited article by Clermont et al. (2013), which described a quadruplex PCR method for assigning *E. coli* isolates to phylogroups A, B1, B2, C, D, E, F, and cryptic clades I to V. Several strains showed bands potentially indicating both phylogroups A and C. In such cases, we used specific primers to distinguish between A and C. None of our isolates were ultimately assigned to phylogroup C. Similarly, specific primers were also used for some strains that showed bands potentially indicating either D and E, or E and *E.* clade I.<br>Phylogroup G and H are relatively newly described phylogroups, where phylogroup G is more closely related to phylogroup B2 [1] and phylogroup H is more related to phylogroup D [2]. As we used the Clermont *et al.* (2013) PCR-based phylotyping method, which classifies only the traditional phylogroups, phylogroups G and H are therefore missing from our analysis.

Results<br>5) Line 235-239: Maybe a supplementary table will help visualize easier these results of distribution patterns	Results<br>5) Line 245: As suggested, a supplementary table (S3 Table) has been provided, showing the details of strain distribution patterns.
**Reviewer #4:** Here, authors have molecularly typed a nearly 3300 E. coli isolates from stools and the intestinal mucosa. Overall, this work is well written, and I have minor comments to help improve this work.<br><br>This work used classical molecular typing tools of a cohort of 46 individuals. Most of the techniques are molecular-based characterization of patterns in MSLT, but authors did not perform DNA sequencing (i.e., sanger or WGS). Many portions of the manuscript invoke the term genetic structure of E. coli strains isolates. I would agree should DNA sequencing not be available; however, current rigor-standard require DNA sequencing to invoke such an interpretation. Please revise entire manuscript and correct accordingly.<br>Title needs to be revised, remove genetic structure, and re-word accordingly.<br><br>Please put upfront why this study follows up prior work in the introduction. This should improve clarity.<br><br>Figure 5 please add the legend of each color in the figure.	**Authors:** Thank you. We have revised the manuscript according to the comments/ suggestions.<br><br>As suggested, we removed the term ‘genetic structure’. Instead, we now use the term ‘population structure’, and this has been revised throughout the manuscript, including in the title.<br><br>As advised, at the beginning of the introduction (line 58-61, page 3), we have briefly explained why this study follows up the prior work, especially the study done by Gordon *et al.* (2015)[3].<br><br>Figure 5: As suggested, we have added the legend for each colour in the figure.

Figure 9: please provide more information of the type of analysis it is not clear to the normal reader.	Figure 9: As suggested, we have revised the figure legend and added more information to make the analysis easier to understand.

References:

1. Clermont O, Dixit OVA, Vangchhia B, Condamine B, Dion S, Bridier-Nahmias A, et al. Characterization and rapid identification of phylogroup G in Escherichia coli, a lineage with high virulence and antibiotic resistance potential. Environmental Microbiology. 2019;21(8):3107-17. doi: https://doi.org/10.1111/1462-2920.14713.
2. Denamur E, Clermont O, Bonacorsi S, Gordon D. The population genetics of pathogenic Escherichia coli. Nature Reviews Microbiology. 2020. doi: 10.1038/s41579-020-0416-x.
3. Gordon DM, O'Brien CL, Pavli P. Escherichia coli diversity in the lower intestinal tract of humans. Environmental Microbiology Reports. 2015;7(4):642-8. doi: 10.1111/1758-2229.12300.
