# Supplementary material for: Diversity, distribution, and population structure of Escherichia coli in the lower gastrointestinal tract of humans
Source: PLoS One. 2025 Jul 10;20(7):e0328147. doi: 10.1371/journal.pone.0328147 (PMC12244825; doi:10.1371/journal.pone.0328147)
Supplement: Attachment — Submitted filename: Response to Reviewers .docx [file pone.0328147.s012.docx]

**Authors’ response to the journal requirements and reviewers’ comments are provided below for PONE-D-24-33067R2.**

We have listed and explained all the changes below, so the revisions are easy to follow. This document includes two tables: 1) authors’ response to the journal requirements, and 2) authors’ response to reviewers’ comments.

**1) Authors’ response to journal requirements**

| **Journal requirements** | **Authors’ response** |
| --- | --- |
| Please review your reference list to ensure that it is complete and correct. If you have cited papers that have been retracted, please include the rationale for doing so in the manuscript text or remove these references and replace them with relevant current references. Any changes to the reference list should be mentioned in the rebuttal letter that accompanies your revised manuscript. If you need to cite a retracted article, indicate the article’s retracted status in the References list and also include a citation and full reference for the retraction notice. | **Authors:** The manuscript does not contain any retracted papers in the reference list. Two citations (citations no. 12 and 13) have been newly included in the manuscript and reference list, as Reviewer #3 suggested adding the missing citations for literature mentioned in lines 68 and 71. |
| Additional Editor Comments (if provided):  Please address the comments of Reviewer 3 so that the paper can be accepted. | **Authors:** We have addressed the comments provided by Reviewer #3 in the following table and revised our manuscript accordingly. |

**2) Authors’ response to reviewers’ comments**

| **Reviewer’s response to questions** | | **Authors’ response** |
| --- | --- | --- |
| 1. If the authors have adequately addressed your comments raised in a previous round of review and you feel that this manuscript is now acceptable for publication, you may indicate that here to bypass the “Comments to the Author” section, enter your conflict of interest statement in the “Confidential to Editor” section, and submit your "Accept"  recommendation. | Reviewer #3: (No Response) | - |
|  | Reviewer #4: All comments have been addressed | **Authors:** Thank you. |
| 2. Is the manuscript technically sound, and do the data support the conclusions?  The manuscript must describe a technically sound piece of scientific research with data that supports the conclusions.  Experiments must have been conducted rigorously, with appropriate controls, replication, and sample sizes. The  conclusions must be drawn appropriately based on the data presented. | Reviewer #3: Yes | **Authors:** Thank you. |
|  | Reviewer #4: Yes |  |
| 3. Has the statistical analysis been performed appropriately and rigorously? | Reviewer #3: Yes | **Authors:** Thank you. |
|  | Reviewer #4: Yes |  |
| 4. Have the authors made all data underlying the findings in their manuscript fully available?  The PLOS Data policy requires authors to make all data underlying the findings described in their manuscript fully available without restriction, with rare exception (please refer to the Data Availability Statement in the manuscript PDF file). The data should be provided as part of the manuscript or its supporting information, or deposited to a public repository. For example, in addition to summary statistics, the data points behind means, medians and variance  measures should be available. If there are restrictions on publicly sharing data—e.g. participant privacy or use of data from a third party—those must be specified. | Reviewer #3: Yes | **Authors:** Thank you. |
|  | Reviewer #4: Yes |  |
| 5. Is the manuscript presented in an intelligible fashion and written in standard English?  PLOS ONE does not copyedit accepted manuscripts, so the language in submitted articles must be clear, correct, and  unambiguous. Any typographical or grammatical errors should be corrected at revision, so please note any specific errors here. | Reviewer #3: Yes | **Authors:** Thank you. |
|  | Reviewer #4: Yes |  |
| 6. Review comments by reviewers and authors’ responses. | | |
| **Reviewer #3:** Thank you to the authors for addressing and give detailed explanation to my previous comments. I have an extra few minor comments that are listed below.  Previously I commented on why C, G and H phylogroups were not included in the study and authors did a very good  job explaining why.  - To avoid future confusion of the readers, explain in a briefly manner, why phylogroup C is not present in the study  - Why the need of using specific primers to distinguish between phylogroup A and C and the others  - And add this. “As we used the Clermont et al. (2013) PCR-based phylotyping method, which classifies only the  traditional phylogroups, phylogroups G and H are therefore missing from our analysis.”  Introduction  - Line 73: Missing reference. … C, E, F, G, and H. [ ]  - Line 76: Missing reference … infections in humans. [ ]  Materials and methods  - Line 145: The PCR step that says elongation should be “annealing”  - Line 163: Please add “which is specific for determining the phylogroups”  - Line 164: Add the word “between” in “Specific primers were used to distinguish between phylogroup ….”  Discussion  - Line 324: Please clarify this sentence “In contrast, the average number of genotypically diverse strains was observed when the dominant strain belonged to phylogroup A, B1, D, and F”. Is the most average number? Higher than the average?  Figures and Tables  - Fig 2: Extra “o” in phylogroups. Both in legend and Y-axis. Also, move the box where the PValue is depicted  elsewhere on the same figure or outside the actual plot because is on top of a 10% value from a phylogroup.  - S2 fig: Eliminate extra “0” decimals from Y-axis  - S3 Table: Explain what is “Nil” | **Authors:** Thank you. We have revised the manuscript according to the reviewer’s comments/ suggestions, which are detailed below.  As suggested, we have included the information in our manuscript.  - We included the information that none of our studied isolates were found to belong to phylogroup C (Line 201).  - We have added information regarding the need of using specific primers to distinguish between phylogroup A and C and the others (Line 161-165).  - To be consistent with the previous sentence, we have slightly paraphrased the sentence as follows- “As the Clermont phylo-typing method classifies only the traditional phylogroups, phylogroups G and H are therefore missing from our analysis” (Line 159-161).  Introduction  - Line 68: We have added the respective references for the information… C, E, F, G, and H. [Ref: 8 and 12]  - Line 71: We have added the respective reference for the information….infections in humans. [Ref: 13]  Materials and methods  - Line 139: Thank you and apologies for the error. We have corrected the word “elongation” to “annealing”.  - Line 158: We have added the words “which is specific” and revised the sentence as suggested.  - Line 162-163: We have added the word “between” and revised the sentence as suggested.  Result  - Line 328-329: We have revised the sentence for better clarification as follows. “In contrast, the number of genotypic strains was found to be relatively higher than the average when the dominant strain belonged to phylogroups A, B1, D, and F.  Figures and Tables  - Fig 2: Thank you and apologies for the error. We have corrected the word “phylogroup” both in legend and Y-axis. Also, we have removed the PValue depicted in the figure, as the value is already mentioned in the manuscript text (Line 207).  - S2 fig: We have eliminated extra “0” decimals from Y-axis.  - S3 Table: We have explained “Nil” in the legend of the S3 Table. ‘Nil’: Indicates that no band was found after the gel electrophoresis, to assign the respective B2 and D isolates as ST73, ST95, ST131 and ST69, respectively, according to Doumith profiling. | |
| **Reviewer #4:** The authors have addressed all my comments. I have no further comments on the paper. The new version is eligible and provides rigor. | **Authors:** Thank you. | |
