# Supplementary material for: Diversity, distribution, and population structure of Escherichia coli in the lower gastrointestinal tract of humans
Source: PLoS One. 2025 Jul 10;20(7):e0328147. doi: 10.1371/journal.pone.0328147 (PMC12244825; doi:10.1371/journal.pone.0328147)
Supplement: Peer review history

# Peer review: Decision Letter 0

**DOI:** 10.1371/journal.pone.0328147.r001

**Authors:** Mehmet Demirci

PONE-D-24-33067

Diversity, distribution, and genetic structure of *Escherichia coli* in the lower gastrointestinal tract of humans

PLOS ONE

Dear Dr. Barua,

Thank you for submitting your manuscript to PLOS ONE. After careful consideration, we have decided that your manuscript does not meet our criteria for publication and must therefore be rejected.

I am sorry that we cannot be more positive on this occasion, but hope that you appreciate the reasons for this decision.

Kind regards,

Mehmet Demirci, PhD

Academic Editor

PLOS ONE

[Note: HTML markup is below. Please do not edit.]

Reviewers' comments:

Reviewer's Responses to Questions

**Comments to the Author**

1. Is the manuscript technically sound, and do the data support the conclusions?

Reviewer #1: Yes

Reviewer #2: Yes

**********

2. Has the statistical analysis been performed appropriately and rigorously?

Reviewer #1: Yes

Reviewer #2: Yes

**********

3. Have the authors made all data underlying the findings in their manuscript fully available?

The PLOS Data policy

Reviewer #1: Yes

Reviewer #2: Yes

**********

4. Is the manuscript presented in an intelligible fashion and written in standard English?

Reviewer #1: Yes

Reviewer #2: Yes

**********

Reviewer #1: Dear Sir

A manuscript entitled: "Microbiological profile of sterile site organisms and culture positivity rates from a

decade of paediatric admissions at a tertiary hospital in South Africa: A lower and

middle-income country perspective" is submitted for publication in the esteemed journal of Plos One. In this manuscript the results of a 10 year survey on blood and CSF cultures from a hospital in South Africa is presented.

At first glance, it seems that the manuscript is informative for scientists and physician in the country, and perhaps neighbor countries, and I do not think that it fulfil the insights of international readers. So I suggest it it is better to consider national journals.

Also, there are some problems that it is better to be addressed.

1- As the title is indicating, the sterile sites are being studied, which I thought that many other sterile sites are also the subject of this research, but it is limited to only blood and CSF cultures.

2- In several parts of the manuscript it is indicated that this is a retrospective study, which we know that it is not a retrospective

3- I cant understand why CoNS, Viridans group streptococci and Micrococcus were considered as contaminants, which we know that in many cases S. epidermidis is a sepsis causing agent and in many countries viridance streptococci are considered as the most important causative agent of endocarditis, which is originated from sepsis

4- There is a strong stress on different wards of the studied hospital, which is totally unattractive for an international reader

5- The discussion is simply a literature review

6- The conclusion was obvious even without the study

Reviewer #2: - Lines 163-166: Language describing isolates from an individual and different strains is slightly confusing, please reword to make easier to understand.

- Line 263: typo

- Fig 2: 2 Typos in Phylogroup

- Can authors comment on any correlation between Fig 5 individual types and antibiotic use

**********

what does this mean? ). If published, this will include your full peer review and any attached files.

If you choose “no”, your identity will remain anonymous but your review may still be made public.

**Do you want your identity to be public for this peer review?** For information about this choice, including consent withdrawal, please see our Privacy Policy

Reviewer #1: **Yes: ** Ramin Abiri

Reviewer #2: No

**********

[NOTE: If reviewer comments were submitted as an attachment file, they will be attached to this email and accessible via the submission site. Please log into your account, locate the manuscript record, and check for the action link "View Attachments". If this link does not appear, there are no attachment files.]

- - - - -

For journal use only: PONEDEC3

# Author comment: Author response to Decision Letter 1

**DOI:** 10.1371/journal.pone.0328147.r002

21 Feb 2025

Response to the reviewers’ comments:

1. Is the manuscript technically sound, and do the data support the conclusions? The manuscript must describe a technically sound piece of scientific research with data that supports the conclusions. Experiments must have been conducted rigorously, with appropriate controls, replication, and sample sizes. The conclusions must be drawn appropriately based on the data presented.

Comment by Reviewer #1: Yes

Comment by Reviewer #2: Yes

Response by Authors: We appreciate the reviewers' positive assessment and valuable feedback. Thank you.

2. Has the statistical analysis been performed appropriately and rigorously?

Comment by Reviewer #1: Yes

Comment by Reviewer #2: Yes

Response by Authors: We appreciate the reviewers' positive assessment and valuable feedback. Thank you.

3. Have the authors made all data underlying the findings in their manuscript fully available? The PLOS Data policy requires authors to make all data underlying the findings described in their manuscript fully available without restriction, with rare exception (please refer to the Data Availability Statement in the manuscript PDF file). The data should be provided as part of the manuscript or its supporting information, or deposited to a public repository. For example, in addition to summary statistics, the data points behind means, medians and variance measures should be available. If there are restrictions on publicly sharing data—e.g. participant privacy or use of data from a third party—those must be specified.

Comment by Reviewer #1: Yes

Comment by Reviewer #2: Yes

Response by Authors: We appreciate the reviewers' positive assessment and valuable feedback. Thank you.

4. Is the manuscript presented in an intelligible fashion and written in standard English? PLOS ONE does not copyedit accepted manuscripts, so the language in submitted articles must be clear, correct, and unambiguous. Any typographical or grammatical errors should be corrected at revision, so please note any specific errors here.

Comment by Reviewer #1: Yes

Comment by Reviewer #2: Yes

Response by Authors: We appreciate the reviewers' positive assessment and valuable feedback. Thank you.

5. Review Comments to the Author

Comment by Reviewer #1:

Dear Sir

A manuscript entitled: "Microbiological profile of sterile site organisms and culture positivity rates from a decade of paediatric admissions at a tertiary hospital in South Africa: A lower and middle-income country perspective" is submitted for publication in the esteemed journal of Plos One. In this manuscript the results of a 10 year survey on blood and CSF cultures from a hospital in South Africa is presented. At first glance, it seems that the manuscript is informative for scientists and physician in the country, and perhaps neighbor countries, and I do not think that it fulfil the insights of international readers. So I suggest it it is better to consider national journals. Also, there are some problems that it is better to be addressed.

1- As the title is indicating, the sterile sites are being studied, which I thought that many other sterile sites are also the subject of this research, but it is limited to only blood and CSF cultures.

2- In several parts of the manuscript it is indicated that this is a retrospective study, which we know that it is not a retrospective

3- I cant understand why CoNS, Viridans group streptococci and Micrococcus were considered as contaminants, which we know that in many cases S. epidermidis is a sepsis causing agent and in many countries viridance streptococci are considered as the most important causative agent of endocarditis, which is originated from sepsis

4- There is a strong stress on different wards of the studied hospital, which is totally unattractive for an international reader

5- The discussion is simply a literature review

6- The conclusion was obvious even without the study

Response by Authors:

Response to Reviewer #1:

We noticed a significant mismatch in the comments provided by Reviewer 1. Reviewer 1’s comments, particularly in this section (point #5), do not align with the content of our manuscript, titled, "Diversity, Distribution, and Genetic Structure of Escherichia coli in the Lower Gastrointestinal Tract of Humans”. Instead, Reviewer 1 references a different manuscript titled, "Microbiological Profile of Sterile Site Organisms and Culture Positivity Rates from a Decade of Paediatric Admissions at a Tertiary Hospital in South Africa: A Lower and Middle-Income Country Perspective", which is unrelated to our study. Reviewer 1’s comments are not applicable to our work and are likely relate to the different manuscript. We believe this may have been an unintentional error.

Comment by Reviewer #2:

Lines 163-166: Language describing isolates from an individual and different strains is slightly confusing,

please reword to make easier to understand.

- Line 263: typo

- Fig 2: 2 Typos in Phylogroup

- Can authors comment on any correlation between Fig 5 individual types and antibiotic use

Response by Authors:

Response to Reviewer #2:

We thank the reviewer for the positive assessment and valuable feedback. We have incorporated the suggestions accordingly.

-Line 163-165: Reworded to make it easier to understand.

-Line 263 and Fig 2: We apologise for being unable to find the typos, as ‘Phylogroup’ or ‘phylogroup’ is commonly used in microbiology literature [1-3].

- Correlation to Fig 5: We have incorporated the suggestions. please refer to the lines 231-242.

Reference:

1. Lagerstrom KM, Hadly EA. Under-Appreciated Phylogroup Diversity of *Escherichia coli* within and between Animals at the Urban-Wildland Interface. Applied and Environmental Microbiology. 2023;89(6):e00142-23. doi: doi:10.1128/aem.00142-23.

2. Kelly M, Hynds P, Brown RS, McDermott K, Petculescu I, Majury AL. The use of E. coli phylogrouping and microbial source tracking (non-species specific, human-specific, bovine-specific bacteroidales markers) to elucidate hydro(geo)logical contamination mechanisms in southeastern Ontario, Canada. Environmental Pollution. 2024;363:125080. doi: https://doi.org/10.1016/j.envpol.2024.125080.

3. Stoppe NdC, Silva JS, Carlos C, Sato MIZ, Saraiva AM, Ottoboni LMM, et al. Worldwide Phylogenetic Group Patterns of Escherichia coli from Commensal Human and Wastewater Treatment Plant Isolates. Frontiers in Microbiology. 2017;8. doi: 10.3389/fmicb.2017.02512.

# Peer review: Decision Letter 1

**DOI:** 10.1371/journal.pone.0328147.r003

**Authors:** Daniel Paredes-Sabja

*Escherichia coli*

Dear Dr. Barua,

Thank you for submitting your manuscript to PLOS ONE. After careful consideration, we feel that it has merit but does not fully meet PLOS ONE’s publication criteria as it currently stands. Therefore, we invite you to submit a revised version of the manuscript that addresses the points raised during the review process.

Please submit your revised manuscript by Jun 15 2025 11:59PM. If you will need more time than this to complete your revisions, please reply to this message or contact the journal office at plosone@plos.org . When you're ready to submit your revision, log on to https://www.editorialmanager.com/pone/ and select the 'Submissions Needing Revision' folder to locate your manuscript file.

- A rebuttal letter that responds to each point raised by the academic editor and reviewer(s). You should upload this letter as a separate file labeled 'Response to Reviewers'.
- A marked-up copy of your manuscript that highlights changes made to the original version. You should upload this as a separate file labeled 'Revised Manuscript with Track Changes'.
- An unmarked version of your revised paper without tracked changes. You should upload this as a separate file labeled 'Manuscript'.

If applicable, we recommend that you deposit your laboratory protocols in protocols.io to enhance the reproducibility of your results. Protocols.io assigns your protocol its own identifier (DOI) so that it can be cited independently in the future. For instructions see: https://journals.plos.org/plosone/s/submission-guidelines#loc-laboratory-protocols . Additionally, PLOS ONE offers an option for publishing peer-reviewed Lab Protocol articles, which describe protocols hosted on protocols.io. Read more information on sharing protocols at https://plos.org/protocols?utm_medium=editorial-email&utm_source=authorletters&utm_campaign=protocols .

We look forward to receiving your revised manuscript.

Kind regards,

Daniel Paredes-Sabja, PhD

Academic Editor

PLOS ONE

Journal Requirements:

When submitting your revision, we need you to address these additional requirements.

1. Please ensure that your manuscript meets PLOS ONE's style requirements, including those for file naming. The PLOS ONE style templates can be found at

https://journals.plos.org/plosone/s/file?id=wjVg/PLOSOne_formatting_sample_main_body.pdf and

https://journals.plos.org/plosone/s/file?id=ba62/PLOSOne_formatting_sample_title_authors_affiliations.pdf

2. Please include your full ethics statement in the ‘Methods’ section of your manuscript file. In your statement, please include the full name of the IRB or ethics committee who approved or waived your study, as well as whether or not you obtained informed written or verbal consent. If consent was waived for your study, please include this information in your statement as well.

3. Please upload a new copy of S1-S4 Figures as the detail is not clear. Please follow the link for more information: "https://blogs.plos.org/plos/2019/06/looking-good-tips-for-creating-your-plos-figures-graphics/"" https://blogs.plos.org/plos/2019/06/looking-good-tips-for-creating-your-plos-figures-graphics/"

Please review your reference list to ensure that it is complete and correct. If you have cited papers that have been retracted, please include the rationale for doing so in the manuscript text, or remove these references and replace them with relevant current references. Any changes to the reference list should be mentioned in the rebuttal letter that accompanies your revised manuscript. If you need to cite a retracted article, indicate the article’s retracted status in the References list and also include a citation and full reference for the retraction notice.

Additional Editor Comments (if provided): Please note that I have acted as a reviewer for this manuscript, and you will find my comments below, under Reviewer 4.

[Note: HTML markup is below. Please do not edit.]

Reviewers' comments:

Reviewer's Responses to Questions

**Comments to the Author**

Reviewer #3: (No Response)

Reviewer #4: (No Response)

**********

2. Is the manuscript technically sound, and do the data support the conclusions?

Reviewer #3: Yes

Reviewer #4: Partly

**********

3. Has the statistical analysis been performed appropriately and rigorously?

Reviewer #3: Yes

Reviewer #4: I Don't Know

**********

4. Have the authors made all data underlying the findings in their manuscript fully available?

The PLOS Data policy

Reviewer #3: Yes

Reviewer #4: No

**********

5. Is the manuscript presented in an intelligible fashion and written in standard English?

Reviewer #3: Yes

Reviewer #4: Yes

**********

Reviewer #3: This paper was well written, and methods and experiments explained in detail. I have minor comments that are listed below.

Introduction

- Line 70-71: missing reference “The main niche for E. coli in humans is the lower region of the gut: the ileum, colon, and rectum [ ]”

- Line 72-73: in the sentence “The isolation of E. coli strains and their characteristics may vary from region to region in the intestinal tract of pigs [20].”, the mentioning of pigs came out of nowhere. Can you rephrase so the transition be smoother between human and pig research

Materials and methods

- Line 147: having E clade twice “…A, B1, B2, D, E, F, and E clade.” Why are clade C, G and H not included?

- Line 148: having E clade twice “…or E and E clade.”

Results

- Line 235-239: Maybe a supplementary table will help visualize easier these results of distribution patterns

Reviewer #4: Here, authors have molecularly typed a nearly 3300 E. coli isolates from stools and the intestinal mucosa. Overall, this work is well written, and I have minor comments to help improve this work.

This work used classical molecular typing tools of a cohort of 46 individuals. Most of the techniques are molecular-based characterization of patterns in MSLT, but authors did not perform DNA sequencing (i.e., sanger or WGS). Many portions of the manuscript invoke the term genetic structure of E. coli strains isolates. I would agree should DNA sequencing not be available; however, current rigor-standard require DNA sequencing to invoke such an interpretation. Please revise entire manuscript and correct accordingly.

Title needs to be revised, remove genetic structure, and re-word accordingly.

Please put upfront why this study follows up prior work in the introduction. This should improve clarity.

Figure 5 please add the legend of each color in the figure.

Figure 9: please provide more information of the type of analysis it is not clear to the normal reader.

**********

what does this mean? ). If published, this will include your full peer review and any attached files.

If you choose “no”, your identity will remain anonymous but your review may still be made public.

**Do you want your identity to be public for this peer review?** For information about this choice, including consent withdrawal, please see our Privacy Policy

Reviewer #3: No

Reviewer #4: No

**********

[NOTE: If reviewer comments were submitted as an attachment file, they will be attached to this email and accessible via the submission site. Please log into your account, locate the manuscript record, and check for the action link "View Attachments". If this link does not appear, there are no attachment files.]

While revising your submission, please upload your figure files to the Preflight Analysis and Conversion Engine (PACE) digital diagnostic tool, https://pacev2.apexcovantage.com/ . PACE helps ensure that figures meet PLOS requirements. To use PACE, you must first register as a user. Registration is free. Then, login and navigate to the UPLOAD tab, where you will find detailed instructions on how to use the tool. If you encounter any issues or have any questions when using PACE, please email PLOS at figures@plos.org

# Author comment: Author response to Decision Letter 2

**DOI:** 10.1371/journal.pone.0328147.r004

21 May 2025

Authors’ response to the journal requirements and reviewers’ comments are provided below for PONE-D-24-33067R2.

We have listed and explained all the changes below, so the revisions are easy to follow. This document includes- 1) our response to the journal requirements/ editor, and 2) our response to each of the reviewer’s comments.

1) Authors’ response to journal requirements/ editor

Journal requirements: Please ensure that your manuscript meets PLoS ONE's style requirements, including those for file naming. The PLoS ONE style templates can be found at

https://journals.plos.org/plosone/s/file?id=wjVg/PLoSOne_formatting_sample_main_body.pdf and

https://journals.plos.org/plosone/s/file?id=ba62/PLoSOne_formatting_sample_title_authors_affiliations.pdf

Authors’ response: We have followed PLoS ONE’s style and requirements in preparing our revised manuscript.

Journal requirements: Please include your full ethics statement in the ‘Methods’ section of your manuscript file. In your statement, please include the full name of the IRB or ethics committee who approved or waived your study, as well as whether or not you obtained informed written or verbal consent. If consent was waived for your study, please include this information in your statement as well.

Authors’ response: Apologies for the oversight, we have now included more details about the ethical committee. The changes have been included in the ‘Materials and methods’ section under the heading ‘Ethics statement’ (Lines: 99-104).

Journal requirements: Please upload a new copy of S1-S4 Figures as the detail is not clear. Please follow the link for more information:

"https://blogs.plos.org/plos/2019/06/looking-good-tips-for-creating-your-plos-figures-graphics/"

https://blogs.plos.org/plos/2019/06/looking-good-tips-for-creating-your-plos-figures-graphics/

Authors’ response: We have revised S1-S3 Figures for better clarity, and the previous S4 Figure has now been presented as S5 Table. In addition, more information has been added to the legends of S1 and S2 Figures.

Journal requirements: Please review your reference list to ensure that it is complete and correct. If you have cited papers that have been retracted, please include the rationale for doing so in the manuscript text, or remove these references and replace them with relevant current references. Any changes to the reference list should be mentioned in the rebuttal letter that accompanies your revised manuscript. If you need to cite a retracted article, indicate the article’s retracted status in the References list and also include a citation and full reference for the retraction notice.

Authors’ response: The manuscript does not contain any retracted papers in the reference list. Some citations (citations no. 17, 18, 19, and 21) have been newly included in the manuscript and reference list, as Reviewer #3 suggested adding the missing citation for literature mentioned in lines: 75-76.

2) Authors’ response to reviewers’ comments

a. Reviewer’s response to question: If the authors have adequately addressed your comments raised in a previous round of review and you feel that this manuscript is now

acceptable for publication, you may indicate that here to bypass the “Comments to the

Author” section, enter your conflict of interest statement in the “Confidential to Editor”

section, and submit your "Accept" recommendation

Reviewer #3: (No Response)

Authors: -

Reviewer #4: (No Response)

Authors: -

b. Reviewer’s response to question: Is the manuscript technically sound, and do the data support the conclusions?

The manuscript must describe a technically sound piece of scientific research with data that supports the conclusions. Experiments must have been conducted rigorously, with appropriate controls, replication, and sample sizes. The conclusions must be drawn appropriately based on the data presented.

Reviewer #3: Yes

Authors: Thank you.

Reviewer #4: Partly

Authors: Thank you. Thank you. We would like to highlight the rigorous approach adopted in our study.

From 46 individuals (originally 50), we analysed a total of 3355 E. coli isolates, which represents a relatively large collection of fresh isolates from individuals. For molecular typing of E. coli isolates, we used a rapid, simple, and highly discriminatory MLVA PCR method. We maintained high standards during the laboratory work and analysed our data rigorously.

We were cautious not to overstate our findings and drew conclusions based on our observations, such as the higher abundance of B2 strains in all gut locations, the lack of variation of phylogroups or the four major STs across gut locations, and the identification of individual types based on strain distribution, and among others. Since our aim was to determine the distribution and diversity of E. coli population structure in the lower gut, this study enabled us to examine both population and sub-population structures of E. coli across different gut locations of the lower gut.

Observing location-specific distribution of strains would not have been possible by analysing faecal samples alone, but our study made this possible by using gut biopsy specimens instead of faecal specimens.

c. Reviewer’s response to question: Has the statistical analysis been performed appropriately and rigorously?

Reviewer #3: Yes

Authors: Thank you.

Reviewer #4: I Don't Know

Authors: Thank you. Actually, the manuscript includes a subsection titled ‘Statistical analysis’ under the ‘Materials and methods’ section (Lines: 163-170).

However, we would like to kindly point out where the statistical analyses were carried out in our study. We used JMP software for statistical analysis of our data and visualization of most of the graphs. We performed statistical analysis of our data wherever applicable, such as analysing unique strains per individual; frequency of unique strains according to gender, age, sex, disease status; distribution of four phylogroups in individuals for antibiotic use; strain richness and diversity; phylogroup and four major sequence types of variation according to gut locations, unique strain heterogeneity, etc.

d. Reviewer’s response to question: Have the authors made all data underlying the findings in their manuscript fully available?

The PLOS Data policy requires authors to make all data underlying the findings described in their manuscript fully available without restriction, with rare exception (please refer to the Data Availability Statement in the manuscript PDF file). The data should be provided as part of the manuscript or its supporting information, or deposited to a public repository. For example, in addition to summary statistics, the data points behind means, medians and variance measures should be available. If there are restrictions on publicly sharing data—e.g. participant privacy or use of data from a third party—those must be specified.

Reviewer #3: Yes

Authors: Thank you.

Reviewer #4: No

Authors: Thank you. We have included our raw data, including the metadata in the supplementary section. For example, Table S1 contains information on individuals number, indication for colonoscopy, and ongoing medication; Table S2 provides information on individuals’ age, sex, disease condition, antibiotic consumption, number of gut regions studied per sample, retrieval of isolates and strain richness; Table S3 includes the raw data on frequency of unique strains isolated from respective gut regions and their classification into phylogroups, four major STs, B2 sub-types, and individual types based on strain distribution.

e. Reviewer’s response to question: Is the manuscript presented in an intelligible fashion and written in standard English?

PLoS ONE does not copyedit accepted manuscripts, so the language in submitted articles must be clear, correct, and unambiguous. Any typographical or grammatical errors should be corrected at revision, so please note any specific errors here.

Reviewer #3: Yes

Authors: Thank you.

Reviewer #4: Yes

Authors: Thank you.

Review Comments by Reviewer #3: This paper was well written, and methods and experiments explained in detail. I have minor comments that are listed below.

Introduction

Line 70-71: missing reference “The main niche for E. coli in humans is the lower region of the gut: the ileum, colon, and rectum [ ]”

Line 72-73: in the sentence “The isolation of E. coli strains and their characteristics may vary from region to region in the intestinal tract of pigs [20].”, the mentioning of pigs came out of nowhere. Can you rephrase so the transition be smoother between human and pig research

Authors: We appreciate this assessment and have revised the manuscript according to the reviewer’s comments/ suggestions, which are detailed below.

Introduction

Line 75-76: We have provided references for the information and slightly paraphrased the sentences.

Line 78-81: As advised, the sentence has been paraphrased. The example of the pig study has been categorized under mammals, which would provide a smoother transition from pig research to human research.

Review Comments by Reviewer #3: Materials and methods

3) Line 147: having E clade twice “…A, B1, B2, D, E, F, and E clade.” Why are clade C, G and H not included?

4) Line 148: having E clade twice “…or E and E clade.”

Authors: Materials and methods

We apologise for the confusion- we have corrected the text and now provided the full form, where E refers to phylogroup E, and E clade refers to the Escherichia clades (please refer to lines 157 and 158). For phylogrouping of our E. coli strains, we followed the highly cited article by Clermont et al. (2013), which described a quadruplex PCR method for assigning E. coli isolates to phylogroups A, B1, B2, C, D, E, F, and cryptic clades I to V. Several strains showed bands potentially indicating both phylogroups A and C. In such cases, we used specific primers to distinguish between A and C. None of our isolates were ultimately assigned to phylogroup C. Similarly, specific primers were also used for some strains that showed bands potentially indicating either D and E, or E and E. clade I.

Phylogroup G and H are relatively newly described phylogroups, where phylogroup G is more closely related to phylogroup B2 [1] and phylogroup H is more related to phylogroup D [2]. As we used the Clermont et al. (2013) PCR-based phylotyping method, which classifies only the traditional phylogroups, phylogroups G and H are therefore missing from our analysis.

Review Comments by Reviewer #3: Results

5) Line 235-239: Maybe a supplementary table will help visualize easier these results of distribution patterns.

Authors: Results

5) Line 247: As suggested, a supplementary table (S3 Table) has been provided, showing the details of strain distribution patterns.

Review Comments by Reviewer #4: Here, authors have molecularly typed a nearly 3300 E. coli isolates from stools and the intestinal mucosa.

Overall, this work is well written, and I have minor comments to help improve this work.

This work used classical molecular typing tools of a cohort of 46 individuals. Most of the techniques are molecular-based characterization of patterns in MSLT, but authors did not perform DNA sequencing (i.e., sanger or WGS). Many portions of the manuscript invoke the term genetic structure of E. coli strains isolates. I would agree should DNA sequencing not be available; however, current rigor-standard require DNA sequencing to invoke such an interpretation. Please revise entire manuscript and correct accordingly.

Title needs to be revised, remove genetic structure, and re-word accordingly.

Authors: Thank you. We have revised the manuscript according to the comments/ suggestions.

As suggested, we removed the term ‘genetic structure’. Instead, we now use the term ‘population structure’, and this has been revised throughout the manuscript, including in the title.

Review Comments by Reviewer #4: Please put upfront why this study follows up prior work in the introduction. This should improve clarity.

Authors: As advised, at the beginning of the introduction (line 58-61, page 3), we have briefly explained why this study follows up the prior work, especially the study done by Gordon et al. (2015)[3].

Review Comments by Reviewer #4: Figure 5 please add the legend of each color in the figure.

Authors: Figure 5: As suggested, we have added the legend for each colour in the figure.

Review Comments by Reviewer #4: Figure 9: please provide more information of the type of analysis it is not clear to the normal reader.

Authors: Figure 9: As suggested, we have revised the figure legend and added more information to make the analysis easier to understand.

References:

1. Clermont O, Dixit OVA, Vangchhia B, Condamine B, Dion S, Bridier-Nahmias A, et al. Characterization and rapid identification of phylogroup G in Escherichia coli, a lineage with high virulence and antibiotic resistance potential. Environmental Microbiology. 2019;21(8):3107-17. doi: https://doi.org/10.1111/1462-2920.14713.

2. Denamur E, Clermont O, Bonacorsi S, Gordon D. The population genetics of pathogenic Escherichia coli. Nature Reviews Microbiology. 2020. doi: 10.1038/s41579-020-0416-x.

3. Gordon DM, O'Brien CL, Pavli P. Escherichia coli diversity in the lower intestinal tract of humans. Environmental Microbiology Reports. 2015;7(4):642-8. doi: 10.1111/1758-2229.12300.

Attachment Submitted filename: Response to Reviewers.pdf

# Peer review: Decision Letter 2

**DOI:** 10.1371/journal.pone.0328147.r005

**Authors:** Daniel Paredes-Sabja

*Escherichia coli*

Dear Dr. Barua,

Thank you for submitting your manuscript to PLOS ONE. After careful consideration, we feel that it has merit but does not fully meet PLOS ONE’s publication criteria as it currently stands. Therefore, we invite you to submit a revised version of the manuscript that addresses the points raised during the review process.

Please submit your revised manuscript by Jul 28 2025 11:59PM. If you will need more time than this to complete your revisions, please reply to this message or contact the journal office at plosone@plos.org . When you're ready to submit your revision, log on to https://www.editorialmanager.com/pone/ and select the 'Submissions Needing Revision' folder to locate your manuscript file.

- A rebuttal letter that responds to each point raised by the academic editor and reviewer(s). You should upload this letter as a separate file labeled 'Response to Reviewers'.
- A marked-up copy of your manuscript that highlights changes made to the original version. You should upload this as a separate file labeled 'Revised Manuscript with Track Changes'.
- An unmarked version of your revised paper without tracked changes. You should upload this as a separate file labeled 'Manuscript'.

If applicable, we recommend that you deposit your laboratory protocols in protocols.io to enhance the reproducibility of your results. Protocols.io assigns your protocol its own identifier (DOI) so that it can be cited independently in the future. For instructions see: https://journals.plos.org/plosone/s/submission-guidelines#loc-laboratory-protocols . Additionally, PLOS ONE offers an option for publishing peer-reviewed Lab Protocol articles, which describe protocols hosted on protocols.io. Read more information on sharing protocols at https://plos.org/protocols?utm_medium=editorial-email&utm_source=authorletters&utm_campaign=protocols .

We look forward to receiving your revised manuscript.

Kind regards,

Daniel Paredes-Sabja, PhD

Academic Editor

PLOS ONE

Journal Requirements:

Please review your reference list to ensure that it is complete and correct. If you have cited papers that have been retracted, please include the rationale for doing so in the manuscript text, or remove these references and replace them with relevant current references. Any changes to the reference list should be mentioned in the rebuttal letter that accompanies your revised manuscript. If you need to cite a retracted article, indicate the article’s retracted status in the References list and also include a citation and full reference for the retraction notice.

Additional Editor Comments (if provided):

Please address the comments of Reviewer 3 so that the paper can be accepted.

[Note: HTML markup is below. Please do not edit.]

Reviewers' comments:

Reviewer's Responses to Questions

**Comments to the Author**

Reviewer #3: (No Response)

Reviewer #4: All comments have been addressed

**********

2. Is the manuscript technically sound, and do the data support the conclusions?

Reviewer #3: Yes

Reviewer #4: Yes

**********

3. Has the statistical analysis been performed appropriately and rigorously?

Reviewer #3: Yes

Reviewer #4: Yes

**********

4. Have the authors made all data underlying the findings in their manuscript fully available?

The PLOS Data policy

Reviewer #3: Yes

Reviewer #4: Yes

**********

5. Is the manuscript presented in an intelligible fashion and written in standard English?

Reviewer #3: Yes

Reviewer #4: Yes

**********

Reviewer #3: Thank you to the authors for addressing and give detailed explanation to my previous comments. I have an extra few minor comments that are listed below.

Previously I commented on why C, G and H phylogroups were not included in the study and authors did a very good job explaining why.

- To avoid future confusion of the readers, explain in a briefly manner, why phylogroup C is not present in the study

- Why the need of using specific primers to distinguish between phylogroup A and C and the others

- And add this. “As we used the Clermont et al. (2013) PCR-based phylotyping method, which classifies only the traditional phylogroups, phylogroups G and H are therefore missing from our analysis.”

Introduction

- Line 73: Missing reference. … C, E, F, G, and H. [ ]

- Line 76: Missing reference … infections in humans. [ ]

Materials and methods

- Line 145: The PCR step that says elongation should be “annealing”

- Line 163: Please add “which is specific for determining the phylogroups”

- Line 164: Add the word “between” in � “Specific primers were used to distinguish between phylogroup ….”

Discussion

- Line 324: Please clarify this sentence “In contrast, the average number of genotypically diverse strains was observed when the dominant strain belonged to phylogroup A, B1, D, and F”. Is the most average number? Higher than the average?

Figures and Tables

- Fig 2: Extra “o” in phylogroups. Both in legend and Y-axis. Also, move the box where the PValue is depicted elsewhere on the same figure or outside the actual plot because is on top of a 10% value from a phylogroup.

- S2 fig: Eliminate extra “0” decimals from Y-axis

- S3 Table: Explain what is “Nil”

Reviewer #4: The authors have addressed all my comments. I have no further comments on the paper.

The new version is elegible and provides rigor

**********

what does this mean? ). If published, this will include your full peer review and any attached files.

If you choose “no”, your identity will remain anonymous but your review may still be made public.

**Do you want your identity to be public for this peer review?** For information about this choice, including consent withdrawal, please see our Privacy Policy

Reviewer #3: No

Reviewer #4: No

**********

[NOTE: If reviewer comments were submitted as an attachment file, they will be attached to this email and accessible via the submission site. Please log into your account, locate the manuscript record, and check for the action link "View Attachments". If this link does not appear, there are no attachment files.]

While revising your submission, please upload your figure files to the Preflight Analysis and Conversion Engine (PACE) digital diagnostic tool, https://pacev2.apexcovantage.com/ . PACE helps ensure that figures meet PLOS requirements. To use PACE, you must first register as a user. Registration is free. Then, login and navigate to the UPLOAD tab, where you will find detailed instructions on how to use the tool. If you encounter any issues or have any questions when using PACE, please email PLOS at figures@plos.org

# Author comment: Author response to Decision Letter 3

**DOI:** 10.1371/journal.pone.0328147.r006

25 Jun 2025

Authors’ response to the journal requirements and reviewers’ comments are provided below for PONE-D-24-33067R2.

We have listed and explained all the changes below, so the revisions are easy to follow. This document includes- 1) authors’ response to the journal requirements, and 2) authors’ response to reviewers’ comments.

1) Authors’ response to journal requirements

Journal requirements: Please review your reference list to ensure that it is complete and correct. If you have cited papers that have been retracted, please include the rationale for doing so in the manuscript text or remove these references and replace them with relevant current references. Any changes to the reference list should be mentioned in the rebuttal letter that accompanies your revised manuscript. If you need to cite a retracted article, indicate the article’s retracted status in the References list and also include a citation and full reference for the retraction notice.

Authors: The manuscript does not contain any retracted papers in the reference list. Two citations (citations no. 12 and 13) have been newly included in the manuscript and reference list, as Reviewer #3 suggested adding the missing citations for literature mentioned in lines 68 and 71.

Additional Editor Comments (if provided):

Please address the comments of Reviewer 3 so that the paper can be accepted.

Authors: We have addressed the comments provided by Reviewer #3 and revised our manuscript accordingly.

2) Authors’ response to reviewers’ comments

1. Reviewer’s response to question: If the authors have adequately addressed your comments raised in a previous round of review and you feel that this manuscript is now acceptable for publication, you may indicate that here to bypass the “Comments to the Author” section, enter your conflict of interest statement in the “Confidential to Editor” section, and submit your "Accept" recommendation.

Reviewer #3: (No Response)

Authors: -

Reviewer #4: All comments have been addressed

Authors: Thank you.

2. Reviewer’s response to question: Is the manuscript technically sound, and do the data support the conclusions?

The manuscript must describe a technically sound piece of scientific research with data that supports the conclusions. Experiments must have been conducted rigorously, with appropriate controls, replication, and sample sizes. The conclusions must be drawn appropriately based on the data presented.

Reviewer #3: Yes

Authors: Thank you.

Reviewer #4: Yes

Authors: Thank you.

3. Reviewer’s response to question: Has the statistical analysis been performed appropriately and rigorously?

Reviewer #3: Yes

Authors: Thank you.

Reviewer #4: Yes

Authors: Thank you.

4. Reviewer’s response to question: Have the authors made all data underlying the findings in their manuscript fully available?

The PLOS Data policy requires authors to make all data underlying the findings described in their manuscript fully available without restriction, with rare exception (please refer to the Data Availability Statement in the manuscript PDF file). The data should be provided as part of the manuscript or its supporting information, or deposited to a public repository. For example, in addition to summary statistics, the data points behind means, medians and variance

measures should be available. If there are restrictions on publicly sharing data—e.g. participant privacy or use of data from a third party—those must be specified.

Reviewer #3: Yes

Authors: Thank you.

Reviewer #4: Yes

Authors: Thank you.

5. Reviewer’s response to question: Is the manuscript presented in an intelligible fashion and written in standard English?

PLOS ONE does not copyedit accepted manuscripts, so the language in submitted articles must be clear, correct, and unambiguous. Any typographical or grammatical errors should be corrected at revision, so please note any specific errors here.

Reviewer #3: Yes

Authors: Thank you.

Reviewer #4: Yes

Authors: Thank you.

Review Comments by Reviewer #3: Thank you to the authors for addressing and give detailed explanation to my previous comments. I have an extra few minor comments that are listed below.

a) Previously I commented on why C, G and H phylogroups were not included in the study and authors did a very good job explaining why.

- To avoid future confusion of the readers, explain in a briefly manner, why phylogroup C is not present in the study

- Why the need of using specific primers to distinguish between phylogroup A and C and the others

- And add this. “As we used the Clermont et al. (2013) PCR-based phylotyping method, which classifies only the traditional phylogroups, phylogroups G and H are therefore missing from our analysis.”

b) Introduction

- Line 73: Missing reference. … C, E, F, G, and H. [ ]

- Line 76: Missing reference … infections in humans. [ ]

c) Materials and methods

- Line 145: The PCR step that says elongation should be “annealing”

- Line 163: Please add “which is specific for determining the phylogroups”

- Line 164: Add the word “between” in “Specific primers were used to distinguish between phylogroup ….”

d) Discussion

- Line 324: Please clarify this sentence “In contrast, the average number of genotypically diverse strains was observed when the dominant strain belonged to phylogroup A, B1, D, and F”. Is the most average number? Higher than the average?

e) Figures and Tables

- Fig 2: Extra “o” in phylogroups. Both in legend and Y-axis. Also, move the box where the PValue is depicted elsewhere on the same figure or outside the actual plot because is on top of a 10% value from a phylogroup.

- S2 fig: Eliminate extra “0” decimals from Y-axis

- S3 Table: Explain what is “Nil”

Authors: Thank you. We have revised the manuscript according to the reviewer’s comments/ suggestions, which are detailed below.

a) As suggested, we have included the information in our manuscript.

- We included the information that none of our studied isolates were found to belong to phylogroup C (Line 201).

- We have added information regarding the need of using specific primers to distinguish between phylogroup A and C and the others (Line 161-165).

- To be consistent with the previous sentence, we have slightly paraphrased the sentence as follows- “As the Clermont phylo-typing method classifies only the traditional phylogroups, phylogroups G and H are therefore missing from our analysis” (Line 159-161).

b) Introduction

- Line 68: We have added the respective references for the information… C, E, F, G, and H. [Ref: 8 and 12]

- Line 71: We have added the respective reference for the information….infections in humans. [Ref: 13]

c) Materials and methods

- Line 139: Thank you and apologies for the error. We have corrected the word “elongation” to “annealing”.

- Line 158: We have added the words “which is specific” and revised the sentence as suggested.

- Line 162-163: We have added the word “between” and revised the sentence as suggested.

d) Result

- Line 328-329: We have revised the sentence for better clarification as follows. “In contrast, the number of genotypic strains was found to be relatively higher than the average when the dominant strain belonged to phylogroups A, B1, D, and F.

e) Figures and Tables

- Fig 2: Thank you and apologies for the error. We have corrected the word “phylogroup” both in legend and Y-axis. Also, we have removed the PValue depicted in the figure, as the value is already mentioned in the manuscript text (Line 207).

- S2 fig: We have eliminated extra “0” decimals from Y-axis.

- S3 Table: We have explained “Nil” in the legend of the S3 Table. ‘Nil’: Indicates that no band was found after the gel electrophoresis, to assign the respective B2 and D isolates as ST73, ST95, ST131 and ST69, respectively, according to Doumith profiling.

Review Comments by Reviewer #4: The authors have addressed all my comments. I have no further comments on the paper. The new version is eligible and provides rigor.

Authors: Thank you.

Attachment Submitted filename: Response to Reviewers .docx

# Editor report: Decision Letter 3

**DOI:** 10.1371/journal.pone.0328147.r007

**Authors:** Daniel Paredes-Sabja

Diversity, distribution, and population structure of *Escherichia coli* in the lower gastrointestinal tract of humans

PONE-D-24-33067R3

Dear Dr. Barua,

We’re pleased to inform you that your manuscript has been judged scientifically suitable for publication and will be formally accepted for publication once it meets all outstanding technical requirements.

Within one week, you’ll receive an e-mail detailing the required amendments. When these have been addressed, you’ll receive a formal acceptance letter and your manuscript will be scheduled for publication.

An invoice will be generated when your article is formally accepted. Please note, if your institution has a publishing partnership with PLOS and your article meets the relevant criteria, all or part of your publication costs will be covered. Please make sure your user information is up-to-date by logging into Editorial Manager at Editorial Manager®  and clicking the ‘Update My Information' link at the top of the page. If you have any questions relating to publication charges, please contact our Author Billing department directly at authorbilling@plos.org.

If your institution or institutions have a press office, please notify them about your upcoming paper to help maximize its impact. If they’ll be preparing press materials, please inform our press team as soon as possible -- no later than 48 hours after receiving the formal acceptance. Your manuscript will remain under strict press embargo until 2 pm Eastern Time on the date of publication. For more information, please contact onepress@plos.org.

Kind regards,

Daniel Paredes-Sabja, PhD

Academic Editor

PLOS ONE

Additional Editor Comments (optional):

Reviewers' comments:

# Editor report: Acceptance letter

**DOI:** 10.1371/journal.pone.0328147.r008

**Authors:** Daniel Paredes-Sabja

PONE-D-24-33067R3

PLOS ONE

Dear Dr. Barua,

I'm pleased to inform you that your manuscript has been deemed suitable for publication in PLOS ONE. Congratulations! Your manuscript is now being handed over to our production team.

At this stage, our production department will prepare your paper for publication. This includes ensuring the following:

* All references, tables, and figures are properly cited

* All relevant supporting information is included in the manuscript submission,

* There are no issues that prevent the paper from being properly typeset

You will receive further instructions from the production team, including instructions on how to review your proof when it is ready. Please keep in mind that we are working through a large volume of accepted articles, so please give us a few days to review your paper and let you know the next and final steps.

Lastly, if your institution or institutions have a press office, please let them know about your upcoming paper now to help maximize its impact. If they'll be preparing press materials, please inform our press team within the next 48 hours. Your manuscript will remain under strict press embargo until 2 pm Eastern Time on the date of publication. For more information, please contact onepress@plos.org.

If we can help with anything else, please email us at customercare@plos.org.

Thank you for submitting your work to PLOS ONE and supporting open access.

Kind regards,

PLOS ONE Editorial Office Staff

on behalf of

Dr. Daniel Paredes-Sabja

Academic Editor

PLOS ONE
